# CD300f:IL-5 cross-talk inhibits adipose tissue eosinophil homing and subsequent IL-4 production

**DOI:** 10.1038/s41598-017-06397-4

**Published:** 2017-07-19

**Authors:** Perri Rozenberg, Hadar Reichman, Israel Zab-Bar, Michal Itan, Metsada Pasmanik-Chor, Carine Bouffi, Udi Qimron, Ido Bachelet, Patricia C. Fulkerson, Marc E. Rothenberg, Ariel Munitz

**Affiliations:** 1 Department of Clinical Microbiology and Immunology, The Sackler School of Medicine, Tel-Aviv University, Ramat Aviv, 69978 Israel; 2 Bioinformatics Unit, George S. Wise Faculty of Life Sciences, Tel Aviv University, Tel Aviv, 64239 Israel; 3 Division of Allergy and Immunology, Cincinnati Children’s Hospital Medical Center, 3333 Burnet Avenue, Cincinnati, OH 45229 USA; 4 Augmanity Nano LTD, Rehovot, Israel

## Abstract

Eosinophils and their associated cytokines IL-4 and IL-5 are emerging as central orchestrators of the immune-metabolic axis. Herein, we demonstrate that cross-talk between the Ig-superfamily receptor CD300f and IL-5 is a key checkpoint that modifies the ability of eosinophils to regulate metabolic outcomes. Generation of *Il5* ^*Tg*^ */Cd300f* ^−/−^ mice revealed marked and distinct increases in eosinophil levels and their production of IL-4 in the white and brown adipose tissues. Consequently, *Il5* ^*Tg*^ */Cd300f* ^−/−^ mice had increased alternatively activated macrophage accumulation in the adipose tissue. *Cd300f* ^−/−^ mice displayed age-related accumulation of eosinophils and macrophages in the adipose tissue and decreased adipose tissue weight, which was associated with decreased diet-induced weight gain and insulin resistance. Notably, *Il5* ^*Tg*^ */CD300f* ^−/−^ were protected from diet-induced weight gain and glucose intolerance. These findings highlight the cross-talk between IL-5 receptor and CD300f as a novel pathway regulating adipose tissue eosinophils and offer new entry points for therapeutic intervention for obesity and its complications.

## Introduction

Eosinophils are bone marrow-derived granulocytes that have been typically studied in the context of type 2 immune responses such as those observed in allergy, asthma and parasite infections^1^. Beyond their traditional role in inflammation, recent data highlight fundamental functions for eosinophils in adipose tissue homeostasis^2, 3^. In fact, a new paradigm has emerged where in the adipose tissue, eosinophils provide IL-4 to sustain the alternatively activated state of resident macrophages which secrete catecholamines to promote metabolic homeostasis^4–6^. Consequently, eosinophil-derived IL-4 is in the center of an immune-metabolic axis that forestalls obesity and glucose tolerance by promoting energy expenditure or by mechanisms yet to be defined^7^. As such, understanding the molecular pathways which regulate the accumulation and activities of adipose tissue eosinophils has potential implications at preventing and/or treating disorders of metabolism such as obesity.

IL-5 is a potent and specific cytokine for the eosinophil lineage and is responsible for their cellular expansion^8^, release from the bone marrow into the circulation^9^ and their survival^10^. Furthermore, IL-5 “primes” eosinophils to potentiate their responses towards a variety of triggers including those mediated by the eotaxin family of chemokines (e.g. CCL11, CCL24, CCL26)^11^. Inhibition of IL-5 with neutralizing antibodies is protective against a variety of eosinophilic diseases and indeed humanized anti-IL-5 antibodies have recently been approved for clinical use in patients with asthma, particularly those with the eosinophilic endotype^12–16^. In mice, adipose tissue eosinophils are readily apparent under baseline conditions and innate lymphoid type 2 cells are a major source for IL-5, which is markedly increased following stimulation with IL-33^4^. Following a high fat diet, *Il5* deficient mice display increased weight gain, adipose tissue weight, fasting glucose levels and exhibit glucose intolerance as well as insulin resistance. Conversely, overexpression of *Il5* results in decreased adipose weight and glucose levels following glucose tolerance testing^3, 4^. With the emergence of a variety of anti-eosinophil therapeutics, there is a timely need to further understand the metabolic role and regulation of eosinophils, particularly those related to IL-5.

The CD300 receptor family is an evolutionary conserved set of type I transmembrane receptors located to chromosome 17 and 11 in human and mice, respectively^17^. The CD300-receptor family members are regulatory receptors that are capable of delivering co-activating or inhibitory signals by binding adaptor molecules such as DNAX-associated protein 10 (DAP10) or phosphatases, respectively^18^. Specifically, CD300a and CD300f have long cytoplasmic tails containing immunoreceptor tyrosine based inhibitory motifs (ITIM) that enable them to suppress cellular activation by recruitment of phosphatases such as SHP-1 and SHP-2^17, 18^. Signaling via CD300f is not confined to phosphatases as CD300f can recruit the p85α subunit of the PI3K signaling pathways as well^19, 20^. Intriguingly, under baseline conditions in the adipose tissue, CD300f is highly expressed by eosinophils, whereas other resident cells such as macrophages and dendritic cells express low levels of CD300f^21^. The findings reported in this manuscript were compelled by our unexpected finding of a gross morphological feature seen in genetically engineered hypereosinophilic mice deficient in *Cd300f*; these mice displayed a prominent rigid humpback, composed of a massive accumulation of eosinophil in the adipose tissue. Accordingly, we aimed to define molecular pathways that regulate the activities of adipose tissue eosinophils with specific emphasis on the cross-talk between CD300f and IL-5 receptor signaling. We report that CD300f expression is regulated by IL-5 and that increased IL-5 levels resulted in distinct accumulation of eosinophils in white and brown adipose tissue, which was histologically characterized by mixed eosinophilic and macrophage/giant cell foci and accompanied by increased IL-4 levels, macrophage and monocyte proliferation, alternatively activated macrophage markers and macrophage-derived CCL24 (eotaxin-2). *Cd300f* ^−/−^ mice showed an age-dependent increase in adipose tissue eosinophils, which was associated with decreased adipose tissue weight as well as decreased weight gain and glucose intolerance following high fat diet, a phenomenon which was markedly augmented in *Il5* ^*Tg*^ */CD300f* ^−/−^ mice. These data identify a novel checkpoint for regulating adipose tissue eosinophils and adipose tissue homeostasis, and have potential implications in understanding and treating metabolic diseases such as obesity and diabetes.

## Results

### CD300f is expressed by eosinophil progenitors

**Figure 1:**
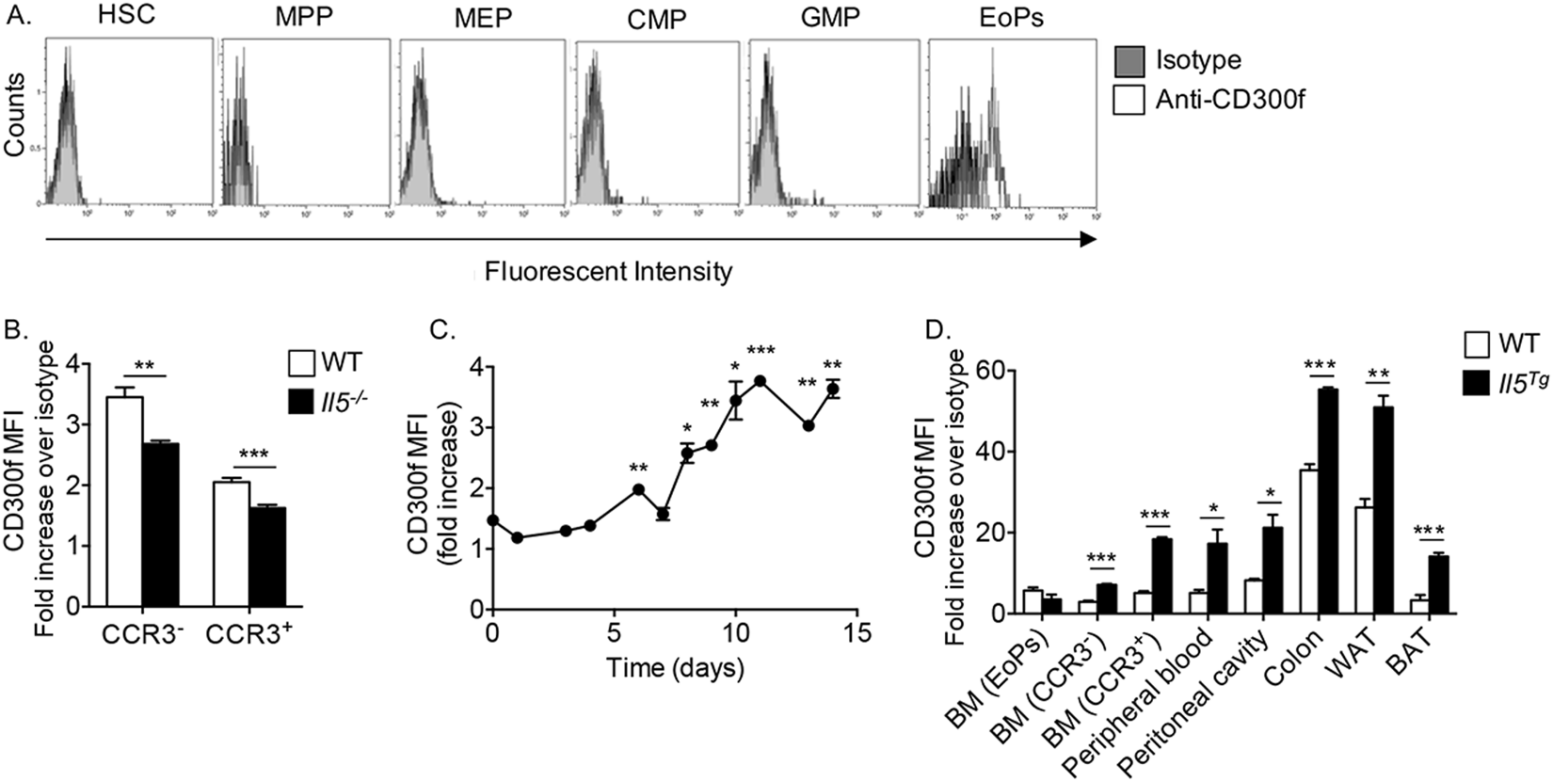
CD300f is expressed by eosinophil progenitors and regulated by IL-5. The expression of CD300f was assessed in bone marrow hematopoietic stem cells (HSCs, CD45^+^/Lin^−^/Sca-1^−^/c-kit^+^/CD135^−^), multipotent progenitors (MPPs, CD45^+^/Lin^−^/Sca-1^−^/c-kit^+^/CD135^−^), myeloid-erythrocyte progenitors (MEPs, CD45^+^/Lin^−^/Sca-1^+^/c-kit^+^/CD34^−^/CD32/16^+^), common myeloid progenitors (CMPs, CD45^+^/Lin^−^/Sca-1^+^/c-kit^+^/CD34^+^/CD32/16^−^), granulocyte-macrophage progenitor (GMPs, CD45^+^/Lin^−^/Sca-1^+^/c-kit^+^/CD34^+^/CD16^+^) and eosinophil progenitors (EoPs, CD45^+^/Lin^−^/Sca-1^+^/c-kit^+^/CD34^int^/IL-5Rα^+^) (**A**). The expression of CD300f was assessed in immature (Siglec-F^+^/CCR3^−^) and mature (Siglec-F^+^/CCR3^+^) bone marrow eosinophils in wild type (WT) and *Il5* ^−/−^ mice (**B**). Bone marrow-derived eosinophils were generated *ex-vivo* and the expression of CD300f on Siglec-F^+^ cells was determined at the indicated time points throughout the eosinophil cell culture (**C**). Finally, the expression of CD300f was assessed in eosinophils (Eos) (**D**), from the indicated organs and in adipose tissue macrophages (Mac), monocytes (Mono) and neutrophils (Neut) (**E**) that were obtained from WT and hypereosinophilic *Il5* transgenic (*Il5* ^*Tg*^) mice (**D**,**E**). In (**E**), the red line indicates fold-increase of 1. Data for (**A**) are representative of n = 5 mice, for (**B**), n = 6 mice, for (**C**), representative of 3–4 independent repeats; for (**D**), n = 3–4 mice; *p < 0.05, **p < 0.01, ***p < 0.001 as analyzed by Student’s *t-test* (**B** and **D**) and two-way ANOVA followed by Tukey post-hoc test (**C**).

We have recently shown that IL-5-induced responses are regulated by ITIM-bearing receptors such as PIR-B^22^. Therefore, we were interested in examining whether bone marrow eosinophil progenitors express additional ITIM-bearing receptors such as those belonging to the CD300 receptor family. To this end, the expression of CD300a and CD300f was determined in hematopoietic stem and progenitor cells including hematopoietic stem cells (HSCs), multipotent progenitors (MPPs), megakaryocyte-erythroid progenitors (MEPs), common myeloid progenitors (CMPs), granulocyte-macrophage progenitors (GMPs) and eosinophil progenitors (EoPs) (see gating strategy in S1)^22, 23^. Among all progenitor cells, the expression of CD300f was exclusive to EoPs (Fig. 1A), while CD300a displayed a broad pattern of expression, readily detected even by early HSCs (S2).

### Overexpression of IL-5 increases CD300f expression

The eosinophil-associated expression pattern of CD300f raised the possibility that the expression of CD300f may be affected by eosinophil-associated cytokines such as IL-5. Indeed, assessment of bone marrow eosinophils from *Il5* ^−/−^ mice demonstrated that the expression of CD300f was decreased in immature (Siglec-F^+^/CCR3^−^) and mature (Siglec-F^+^/CCR3^+^) eosinophils (Fig. 1B). Furthermore, the expression of CD300f steadily increased upon addition of IL-5 to *in vitro* generated bone marrow-derived eosinophils (Fig. 1C, days 1–7 gating on all cells; days 8–14 gating on Siglec-F^+^ cells). Consistent with this finding, the expression of CD300f (but not CD300a, S2) was highly increased on eosinophils that were obtained from IL-5 overexpressing mice (*Il5* ^*Tg*^ mice, Fig. 1D). Interestingly, although the expression of CD300f was highest in colonic and white adipose tissue eosinophils in wild type mice, the expression of CD300f was further increased following exposure to IL-5 (Fig. 1D). Importantly, the specificity of our anti-CD300f antibody was recently established using *Cd300f* ^−/−^ cells^21^.

### IL-5-driven adipose tissue eosinophil accumulation is negatively regulated by CD300f

**Figure 2:**
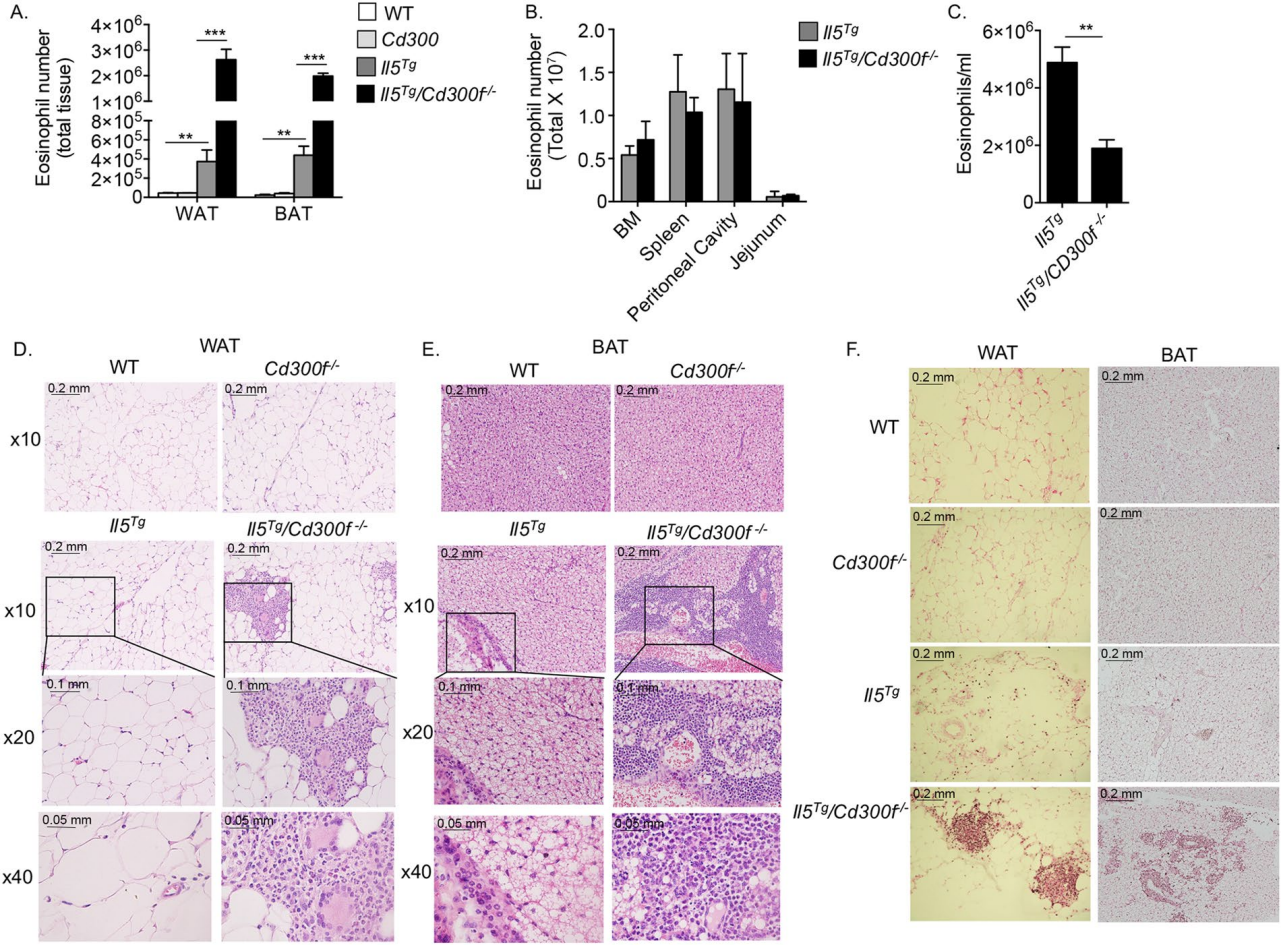
IL-5-driven adipose tissue eosinophil accumulation is negatively regulated by CD300f. The number of total eosinophils was assessed in the white adipose tissue (WAT) and brown adipose tissue (BAT) of six to eight-week old wild type (WT), *Cd300f* ^−/−^, *Il5* ^*Tg*^ and *Il5* ^*Tg*^ */Cd300f* ^−/−^ mice (**A**). In addition, eosinophil levels were enumerated in the bone marrow (BM), spleen, peritoneal cavity, jejunum (**B**) and peripheral blood (**C**) of *Il5* ^*Tg*^ and *Il5* ^*Tg*^ */Cd300f* ^−/−^ mice (**B**). The WAT (**D**, **F**) and BAT (**E**,**F**) of wild type (WT), *Cd300f* ^−/−^, *Il5* ^*Tg*^ and *Il5* ^*Tg*^ */Cd300f* ^−/−^ mice was obtained and subjected to H&E staining (**D**,**E**) as well as anti-eosinophil major basic protein (MBP) stain (**F**). Data for (**A**–**C**) are from n = 5 mice. In (**D**), representative photomicrographs are shown; **p < 0.01, ***p < 0.001 as analyzed by Student’s *t-test* (**A** and **C**).

The finding that CD300f expression was regulated by IL-5 levels raised the hypothesis that CD300f regulates IL-5-induced responses in eosinophils. To assess this possibility, we generated *Il5* ^*Tg*^ mice that lack *Cd300f* (*Il5* ^*Tg*^ */Cd300f* ^−/−^ mice). Strikingly, six to eight week old *Il5* ^*Tg*^ */Cd300f* ^−/−^ mice developed an obvious rigid humpback, which was anatomically located in the proximity of the interscapular brown adipose tissue (BAT)^24^. Thus, we were interested in determining whether *Il5* ^*Tg*^ */Cd300f* ^−/−^ mice display increased eosinophil accumulation in the BAT. Eosinophils were readily detected in the BAT of *Il5* ^*Tg*^ mice. Nonetheless, *Il5* ^*Tg*^ */Cd300f* ^−/−^ mice displayed substantially elevated BAT eosinophil levels (~5-fold increase) in comparison with *Il5* ^*Tg*^ mice (Fig. 2A). To assess whether increased eosinophilia in *Il5* ^*Tg*^ */Cd300f* ^−/−^ mice was restricted to the BAT or evident in the white adipose tissue (WAT) as well, the inguinal white adipose tissues of *Il5* ^*Tg*^ */Cd300f* ^−/−^ mice were excised and eosinophil levels assessed. The WAT of *Il5* ^*Tg*^ */Cd300f* ^−/−^ mice displayed a ~7.5-fold increase in eosinophil levels in comparison with *Il5* ^*Tg*^ mice (Fig. 2A). Of even greater interest was the finding that elevated eosinophilia was specific to the adipose tissue since bone marrow, splenic and gastrointestinal eosinophil levels were comparable between *Il5* ^*Tg*^ and *Il5* ^*Tg*^ */Cd300f* ^−/−^ mice (Fig. 2B). The increased eosinophil levels in adipose tissue was associated with decreased peripheral blood eosinophilia (Fig. 2C), consistent with a recruitment from the blood into the adipose tissue^21^.

Histological assessment of WAT and BAT revealed marked cellular infiltrate in the adipose tissue of *Il5* ^*Tg*^ */Cd300f* ^−/−^ mice (Fig. 2D,E). In fact, the adipose tissue of *Il5* ^*Tg*^ */Cd300f* ^−/−^ mice displayed numerous cellular foci that consisted of cells with eosinophil, macrophage and giant cell morphology (Fig. 2D,E). Indeed, anti-eosinophil major basic (MBP) protein staining, which specifically stains eosinophils^25^, demonstrated the abundance of eosinophils in these cellular foci (Fig. 2F).

### CD300f negatively regulates eosinophil-dependent adipose tissue IL-4 production

**Figure 3:**
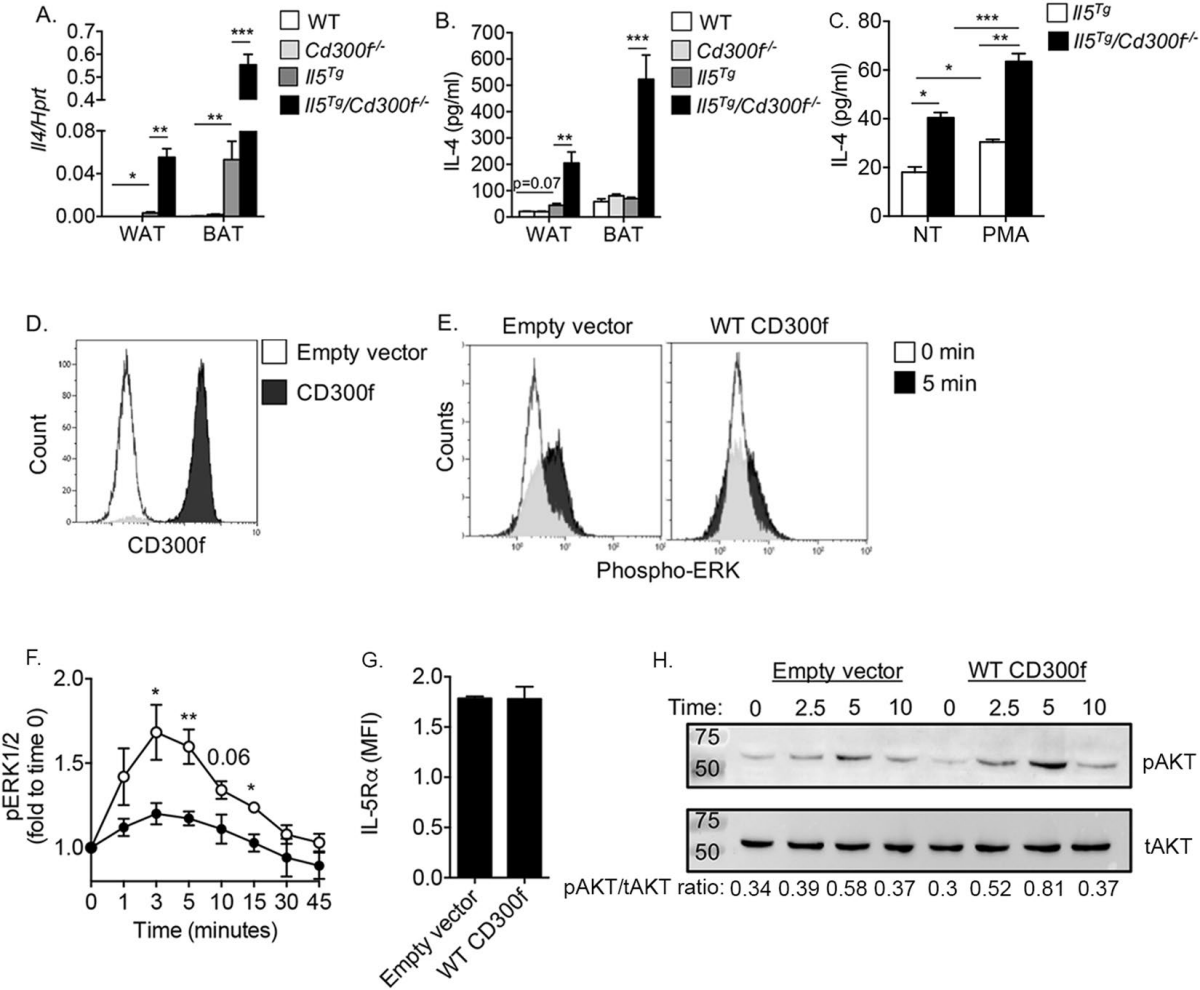
CD300f negatively regulates eosinophil-derived IL-4 production and governs IL-5-induced ERK and pAKT phosphorylation. The mRNA (**A**) expression of IL-4 was assessed in the white and brown adipose tissue (WAT and BAT, respectively) by quantitative PCR and normalized to the house keeping gene hypoxanthine-guanine phosphoribosyltransferase (*Hprt*). Protein expression of IL-4 (**B**) was determined by ELISA. In (**C**), primary eosinophils were purified from the WAT of *Il5* ^*Tg*^ and *Il5* ^*Tg*^ */Cd300f* ^−/−^ mice and either left untreated (NT) or activated with phorbol 12- myristate 13-acetate (PMA). Thereafter, secretion of IL-4 was determined in the culture supernatants (**C**). The expression of CD300f (**D**) and IL-5 receptor α (IL-5Rα) (**G**) in I.29 B cells is shown following viral infection with empty vector or CD300f-containing vector (**D**). Following retroviral infection with empty vector of CD300f-containg vector, I.29 cells were stimulated with IL-5 for the indicated time points (**E**,**F**) and the phosphorylation of ERK (p42/44) (**E**,**F**) and AKT (**H**) was assessed by phosphoflow (**E**,**F**) and western blot, respectively (**H**). Data in (**A**,**B**) are from n = 5 mice, in (**C**) from n = 3 independent experiments, in (**D**,**H**) from n = 4 independent experiments; *p < 0.05, **p < 0.01, ***p < 0.001 as analyzed by Student’s *t-test* (**A**–**C**) and two-way Anova followed by Tukey post-hoc test (**F**).

The unique structural change in the adipose tissue with the presence of macrophages and giant cells suggested that *Il5* ^*Tg*^ */Cd300f* ^−/−^ mice would display increased IL-4 expression^26^. While IL-4 mRNA expression was nearly undetectable in the WAT and BAT of wild type and *Cd300f* ^−/−^ mice in comparison with *Hprt* (0.000076 ± 0.000087 and 0.00017 ± 0.00086, respectively) the expression of IL-4 was markedly increased in the WAT and BAT of *Il5* ^*Tg*^ mice (~69- and ~122-fold increase in comparison with wild type mice). IL-4 mRNA expression was further elevated in the WAT and BAT of *Il5* ^*Tg*^ */Cd300f* ^−/−^ mice by ~17- and ~10-fold compared to *Il5* ^*Tg*^ mice, respectively (Fig. 3A). Elevated IL-4 expression in *Il5* ^*Tg*^ */Cd300f* ^−/−^ mice was observed in adipose tissue protein extracts as well (Fig. 3B).

Since eosinophils are the major source for IL-4 in the adipose tissue^3, 5^, increased IL-4 expression in *Il5* ^*Tg*^ */Cd300f* ^−/−^ mice led us to hypothesize that CD300f may act as a negative regulator of eosinophil-derived IL-4 production. To assess this hypothesis, primary eosinophils were purified from the WAT of *Il5* ^*Tg*^ and *Il5* ^*Tg*^ */Cd300f* ^−/−^ mice and baseline secretion of IL-4 was assessed (Fig. 3C). In agreement with previous studies showing that adipose tissue eosinophils secrete IL-4^2–4, 6, 27^, baseline IL-4 secretion was detected in the supernatants of primary adipose tissue eosinophils that were obtained from *Il5* ^*Tg*^ mice (Fig. 3C). Notably, eosinophils from *Il5* ^*Tg*^ */Cd300f* ^−/−^ mice displayed enhanced secretion of IL-4 under baseline conditions (Fig. 3C). Furthermore, activation of eosinophils from *Il5* ^*Tg*^ */Cd300f* ^−/−^ mice with phorbol 12- myristate 13-acetate (PMA), a potent activator of eosinophils, resulted in substantially increased IL-4 secretion in comparison with PMA-stimulated eosinophils from *Il5* ^*Tg*^ mice and untreated eosinophils (Fig. 3C). Increased eosinophil levels (Fig. 2) and IL-4 production (Fig. 3A-C) by eosinophils raised the possibility that CD300f may regulate eosinophil differentiation and/or maturation in the bone marrow. To address this possibility, GeneChip® microarray analysis of highly purified primary bone marrow eosinophils that were sorted from *Il5* ^*Tg*^ *and Il5* ^*Tg*^ */Cd300f* ^−/−^ mice was performed. This analysis revealed that with the exception of *Cd300f* (*p* < *0.0001*), no other differences were apparent between eosinophils from *Il5* ^*Tg*^ *and Il5* ^*Tg*^ */Cd300f* ^−/−^ mice including no difference in *Il4* mRNA expression (Figure S3). Furthermore, no developmental differences were observed in the absence of *Cd300f* since eosinophils from bone marrow-derived cultures did not show any differences in overall cell counts, cell proliferation or apoptosis (S4). Collectively, this data suggests that increased IL-4 production in the adipose tissue of *Il5* ^*Tg*^ */Cd300f* ^−/−^ mice may be due to increased eosinophil accumulation and/or increased production of IL-4 in *Cd300f* ^−/−^ eosinophils in response to local activating factors.

### CD300f regulates IL-5-induced ERK and AKT phosphorylation

The finding that *Cd300f* ^−/−^ mice displayed markedly increased eosinophil accumulation and subsequent activation in the adipose tissue in the presence of increased IL-5 levels, suggested that CD300f can regulate IL-5-induced signaling in eosinophils. To assess this possibility, mouse I.29 B cells, which are responsive to IL-5^28^, but display no baseline expression of CD300f were used to generate a stable cell line overexpressing CD300f (Fig. 3D). Thereafter, I.29 cells were stimulated with IL-5 and phosphorylation of ERK was determined using PhosphoFlow as we previously published^29–32^. IL-5 stimulated I.29 cells containing an empty vector displayed elevated ERK phosphorylation (Fig. 3E,F). Overexpression of CD300f blocked IL-5-induced ERK activation (Fig. 3E,F). Importantly, decreased IL-5-induced responses in CD300f-expressing I.29 cells were not due to alterations in IL-5Rα expression (Fig. 3G). CD300f has been shown to associate with PI3K^19, 20^. Thus, we were interested in determining whether CD300f could regulate the activation of AKT, a downstream mediator of PI3K signaling^33^. To this end, CD300f-expressing and empty vector infected control cells were stimulated with IL-5 and the phosphorylation of AKT was determined. In response to stimulation with IL-5, CD300f-expressing I.29 cells showed elevated AKT activation in comparison with control cells (Fig. 3H).

### CD300f-IL-5 receptor cross talk regulates alternatively activated macrophage formation in adipose tissue

**Figure 4:**
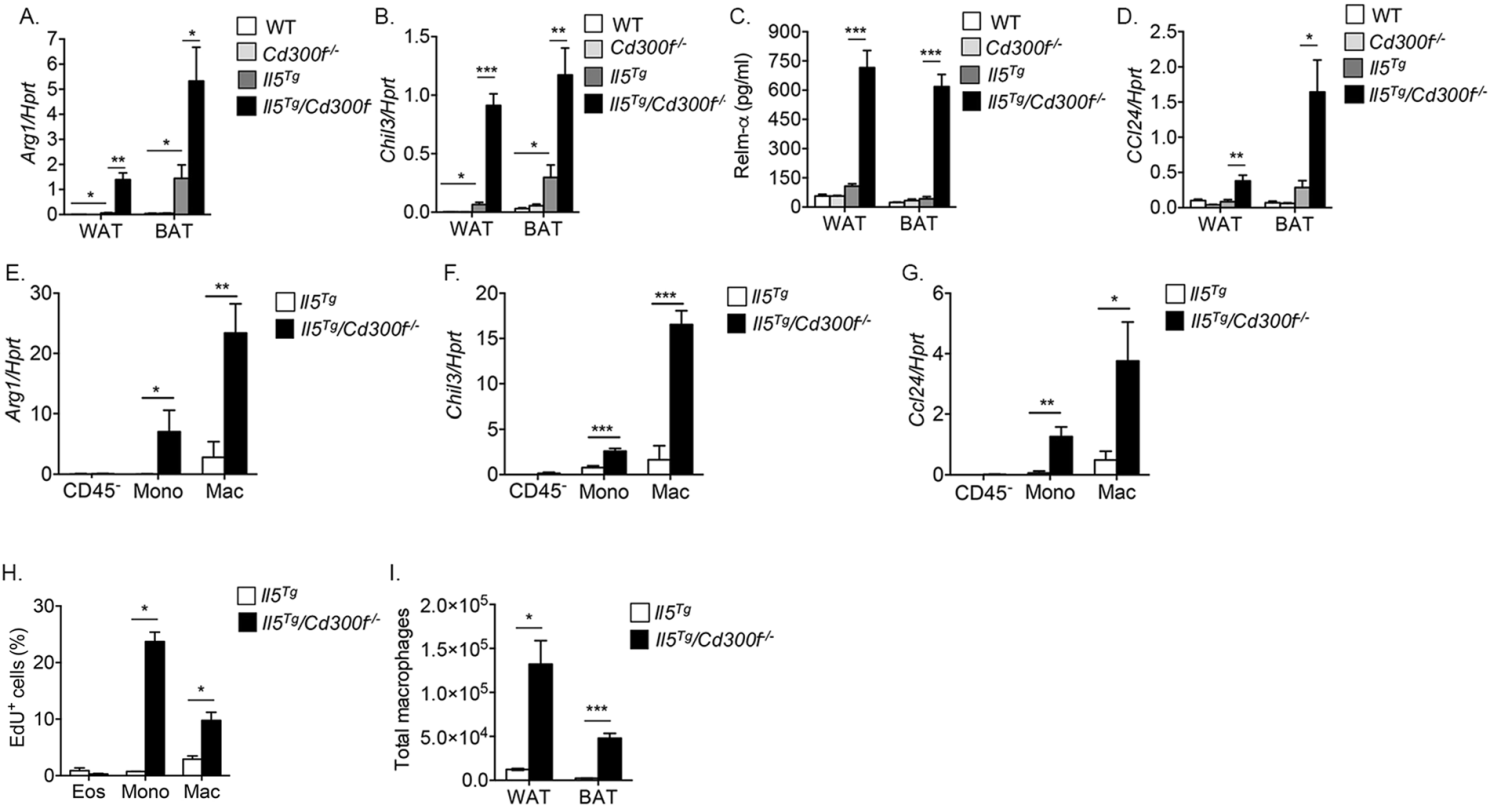
CD300f-IL-5 receptor cross talk regulates alternatively activated macrophage formation in adipose tissue. The expression of hallmark alternatively activated macrophage markers such as *Arg1*, *Chi3l*, Relm-α and *Ccl24* were determined in the adipose tissue of wild type (WT), *Cd300f* ^−/−^, *Il5* ^*Tg*^ and *Il5* ^*Tg*^ */Cd300f* ^−/−^ mice by quantitative PCR and normalized to the house keeping gene hypoxanthine-guanine phosphoribosyltransferase (*Hprt*) (**A**,**B** and **D**) and ELISA (**C**). Furthermore, the expression of *Arg1*, *Chi3l*, and *Ccl24* was determined in the primary adipose tissue macrophages that were sorted from *Il5* ^*Tg*^ and *Il5* ^*Tg*^ */Cd300f* ^−/−^ mice by quantitative PCR (**E**–**G**). *In situ* proliferation of adipose tissue eosinophils, macrophages and monocytes was assessed in *Il5* ^*Tg*^ and *Il5* ^*Tg*^ */Cd300f* ^−/−^ mice using flow cytometric analysis of EdU^+^ cells (**H**). Thereafter, total WAT and BAT macrophage levels in *Il5* ^*Tg*^ and *Il5* ^*Tg*^ */Cd300f* ^−/−^ mice were assessed (**I**). Data in (**A**–**D**) are from n = 5–6 mice; in (**E**–**G**) n = 3; in (**H**,**I**) from n = 6; *p < 0.05, **p < 0.01, ***p < 0.001 as analyzed by Student’s *t-test* (**A**–**I**).

To determine whether increased IL-4 production in the adipose tissue of *Il5* ^*Tg*^ */Cd300f* ^−/−^ mice resulted in increased alternatively activated macrophage formation, the expression of various alternatively activated markers was examined^34^. Indeed, the expression of *Arg1, Chil3l, Ccl24* and Relm-α was markedly increased in the WAT and BAT of *Il5* ^*Tg*^ */Cd300f* ^−/−^ mice in comparison to their expression in wild type, *Cd300f* ^−/−^ and *Il5* ^*Tg*^ mice (Fig. 4A–D). To definitely demonstrate that macrophages were the cellular source of the aforementioned mediators, CD45^−^ cells (representing adipose structural cells such as adipocytes, fibroblasts and endothelial cells), monocytes (CD11b^+^/Ly6c^+^/F4/80^−^/Ly6G^−^/Siglec-F^−^) and macrophages (CD11b^+^/F4/80^+^/Ly6c^−^/Ly6G^−^) were sorted from the WAT of *Il5* ^*Tg*^ and *Il5* ^*Tg*^ */Cd300f* ^−/−^ mice and *Arg1, Chil3* and *Ccl24* expression was determined by qPCR. Macrophages from the WAT of *Il5* ^*Tg*^ */Cd300f* ^−/−^ mice had substantially increased expression of *Arg1*, *Chil3* and *Ccl24* (Fig. 4E–G), in comparison with macrophages from *Il5* ^*Tg*^ mice. Interestingly, monocytes from *Il5* ^*Tg*^ */Cd300f* ^−/−^ displayed induction of *Arg1*, *Chil3* and *Ccl24* as well (Fig. 4E–G), albeit at lower levels than adipose tissue macrophages. CD45^−^ cells showed no expression of the aforementioned molecules (Fig. 4E–G).

Increased IL-4 production in the WAT *Il5* ^*Tg*^ */Cd300f* ^−/−^ mice was associated with enhanced monocyte (defined as: CD45^+^/CD11b^+^/Ly6C^+^/Ly6G^−^ cells) proliferation (Fig. 4H) and to lesser extent macrophages (defined as CD45^+^/CD11b^+^/F480^+^/Ly6C^−^/Ly6G^−^ cells, Fig. 4H). While ~15% of the monocytes from the WAT of *Il5* ^*Tg*^ mice stained positive for EdU, ~37% of the monocytes from the WAT of *Il5* ^*Tg*^ */Cd300f* ^−/−^ mice were EdU^+^ (more than 2-fold increase, p < 0.05). Subsequently, a ~15- and ~10-fold increase in total macrophage numbers was observed in the WAT and BAT of *Il5* ^*Tg*^ */Cd300f* ^−/−^ mice, respectively (Fig. 4I). Since increased numbers of adipose tissue macrophages could result from increased chemotaxis of monocytes and their subsequent differentiation into macrophages, we determined the levels of CCL2, a major monocyte chemoattractant, which may be produced by multiple cell types including eosinophils and fibroblasts in response to GM-CSF^35, 36^. Interestingly, *Il5* ^*Tg*^ */Cd300f* ^−/−^ mice displayed increased *Ccl2* expression in the BAT but not WAT (S5).

### Viable adipose tissue endothelial cells express a CD300f ligand

**Figure 5:**
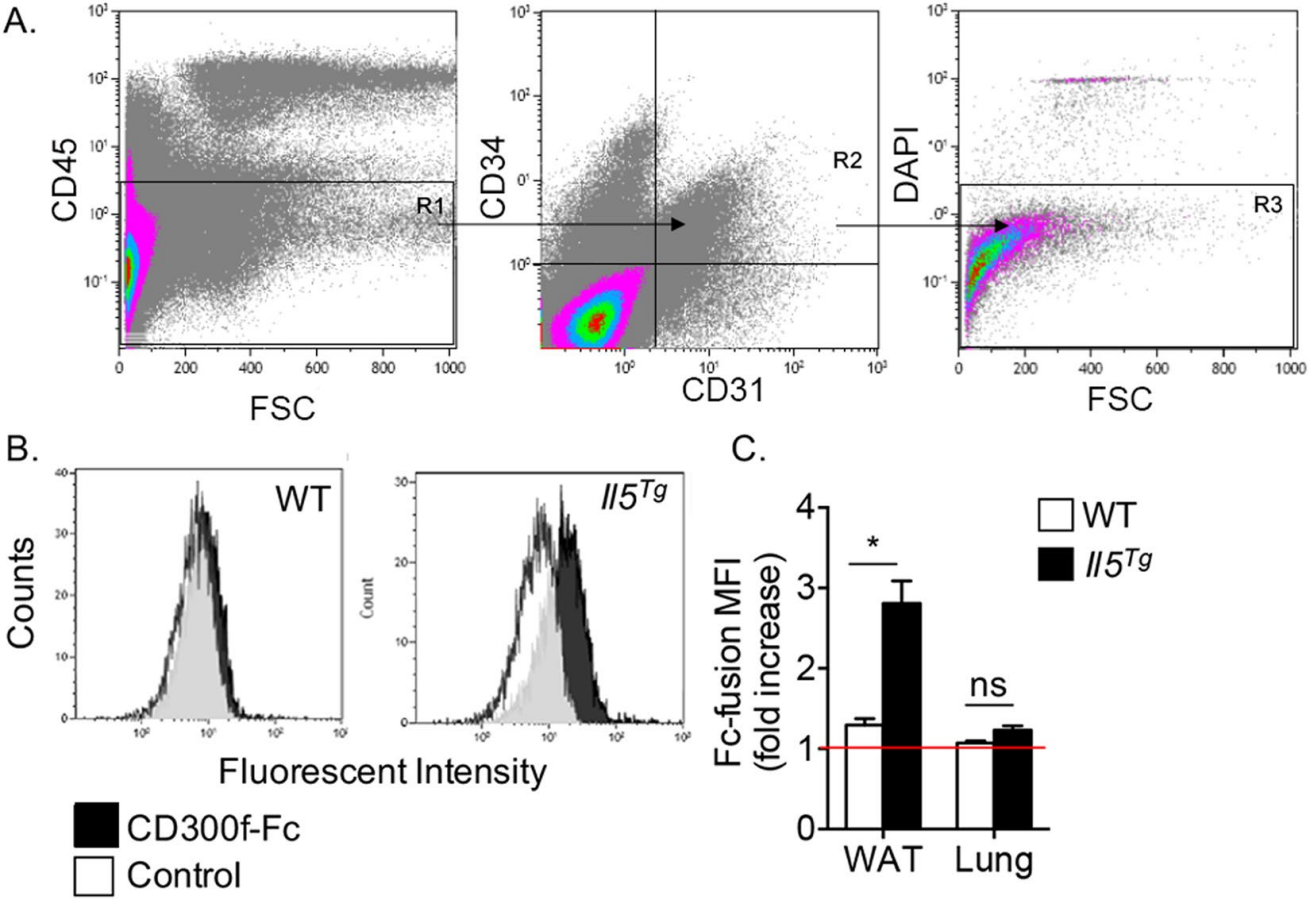
Viable adipose tissue endothelial cells express a CD300f ligand. White adipose tissue (**A**–**D**) and lungs (**D**) from wild type (WT) and *Il5* ^*Tg*^ mice were obtained, enzymatically digested and stained with viability and endothelial cells markers (**A**). Thereafter, the binding of CD300f-IgG1 Fc fusion protein to viable adipose tissue (**B**,**C**) and lung (**D**) endothelial cells was determined by flow cytometry. Data are representative of n = 4 independent experiments; *p < 0.05, ns- non-significant as analyzed by Student’s *t-test* (**C**).

The finding that CD300f specifically regulated the accumulation of eosinophils into the adipose tissue suggested that CD300f ligands may be expressed and perhaps restricted to adipose tissue and that their expression may be augmented by IL-5. To this end, enzymatically digested adipose tissue cells were stained with CD300f-Fc fusion protein. Among all viable cells, CD300f-Fc fusion protein was found to bind endothelial cells (CD45^−^/CD31^+^/CD34^+^/DAPI^−^, Fig. 5A). Notably, binding of CD300f-Fc fusion protein to viable endothelial cells was further augmented in endothelial cells obtained from *Il5* ^*Tg*^ mice (Fig. 5B,C). Importantly, CD300f-Fc fusion protein was specific to viable endothelial cells in the adipose tissue since viable lung endothelial cells displayed nearly no binding (Fig. 5C).

### *Cd300f*^−/−^ mice display age-related accumulation of eosinophils and macrophages

**Figure 6:**
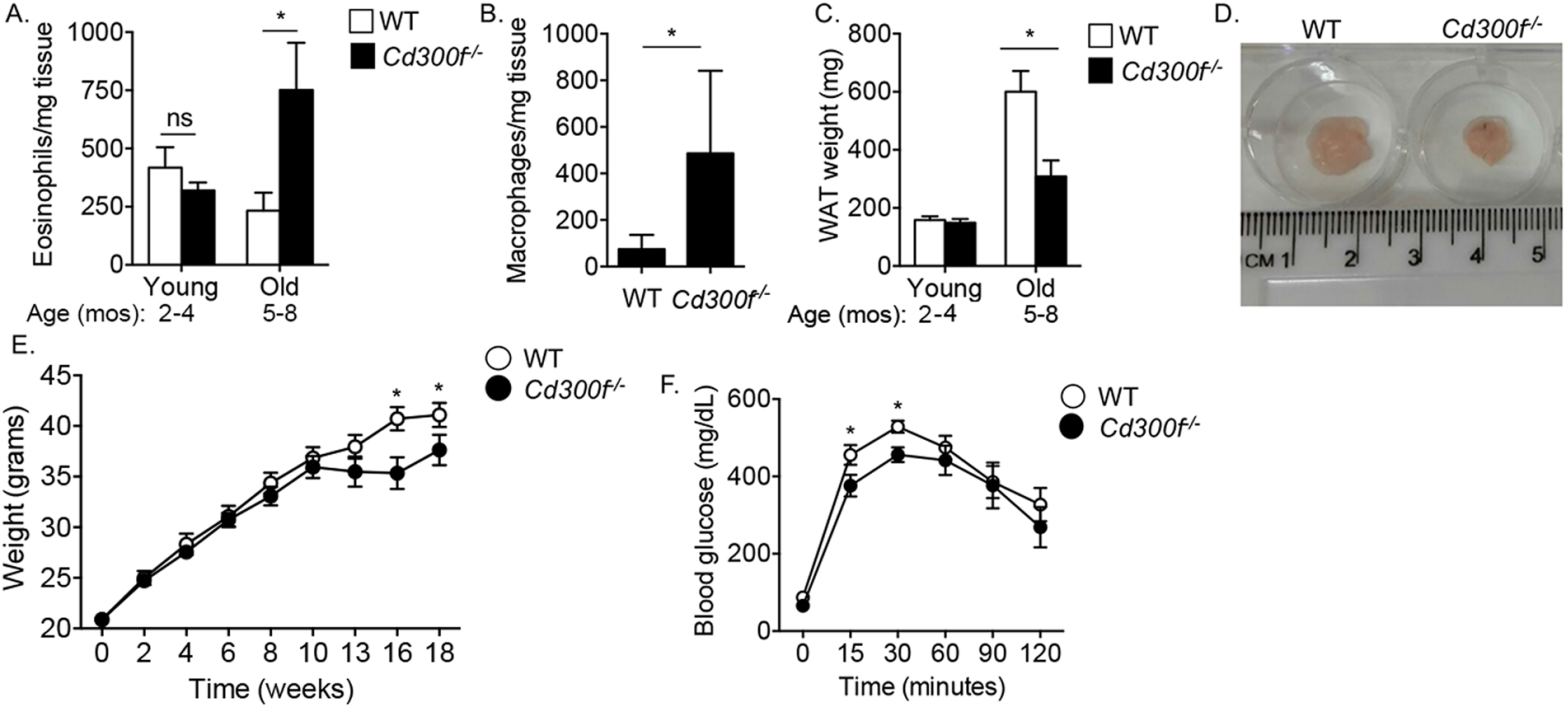
*Cd300f* ^−/−^ mice display age-related accumulation of eosinophils and macrophages and decreased adipose tissue weight. White adipose tissue (WAT) of young (2–4 months) and aged (5–8 months) wild type (WT) and *Cd300f* ^−/−^ mice were obtained and total eosinophils (**A**) and macrophages (**B**) per mg tissue were determined. Moreover, the weight of WATs from WT and *Cd300f* ^−/−^ mice was determined (**C**). In (**D**) a representative photograph of WAT size from WT and *Cd300f* ^−/−^ mice is shown. WT and *Cd300f* ^−/−^ mice were fed with high fat diet (HFD) for up to 18 weeks and weighed at the indicated time points (**E**). Following 18 weeks of HFD, mice were tested for glucose tolerance (**F**). Data in (**A**–**C**) are from n = 3, 2–3 mice per time point; in (**D**) data are representative of on one out of eight; in (**E**–**F**) data are from n = 3 using 10 mice per group; *p < 0.05, **p < 0.01, ***p < 0.001 as analyzed by Student’s *t-test* (**A**–**C**) and two-way ANOVA followed by Tukey post-hoc test (**E**,**F**).

Since CD300f regulated eosinophil homing to the adipose tissue, we were interested in determining whether *Cd300f* ^−/−^ mice displayed increased eosinophil and macrophage levels under baseline conditions. *Cd300f* ^−/−^ mice displayed age-associated accumulation of eosinophils in the adipose tissue (Fig. 6A). Eosinophil levels in *Cd300f* ^−/−^ mice were significantly higher in mice as they achieved 5 months of age. Consistently, increased eosinophil numbers in aged *Cd300f* ^−/−^ mice were associated with increased macrophage numbers (Fig. 6B), as well as decreased WAT weight (Fig. 6C) and size (Fig. 6D).

### *Cd300f*^−/−^ mice display decreased diet-induced weight gain and glucose intolerance

To assess the physiological role of CD300f in obesity and glucose metabolism, eight-week old *Cd300f* ^−/−^ mice were fed with normal chow or high fat diet and their weight as well as sensitivity to glucose challenges were monitored. *Cd300f* ^−/−^ mice displayed decreased weight gain in response to high fat diet that was evident at later (starting at week 13) time points of the diet (Fig. 6E). Decreased weight gain in *Cd300f* ^−/−^ mice was not due to decreased food intake since *Cd300f* ^−/−^ mice that were assessed in metabolic cages displayed slightly but significantly increased appetite (S6). Moreover, following 18 weeks (i.e. when the mice were 26 weeks old) of high fat diet, *Cd300f* ^−/−^ mice displayed increased glucose tolerance (Fig. 6F). No differences were observed in weight gain or glucose sensitivity in response to normal chow diet (S7).

### *Il5*^*Tg*^*/CD300f*^−/−^ are protected from diet-induced weight gain and glucose intolerance

**Figure 7:**
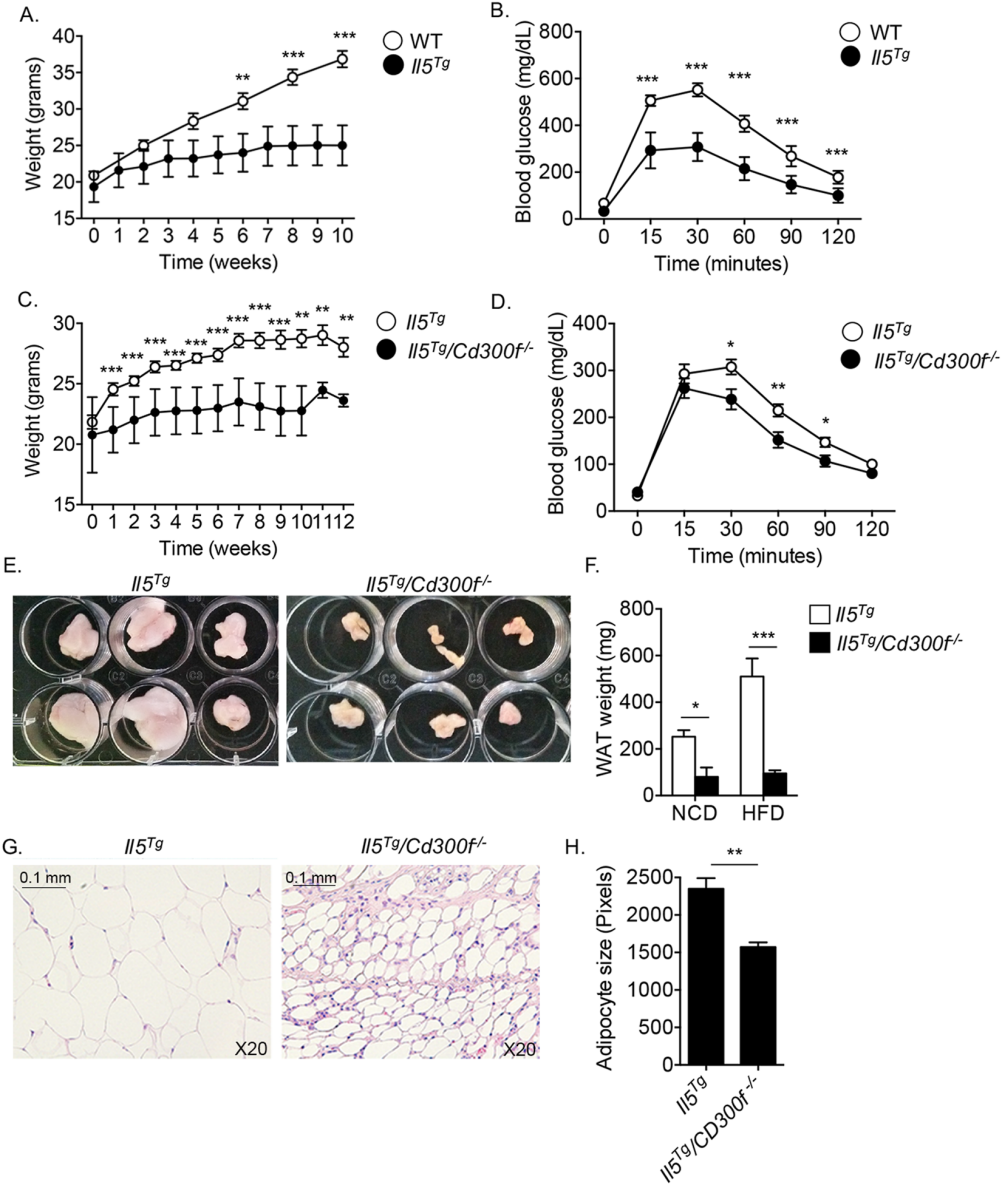
*Il5* ^*Tg*^ */CD300f* ^−/−^ mice are protected from diet-induced weight gain and glucose intolerance. Wild type (WT), *Il5* ^*Tg*^ and *Il5* ^*Tg*^ */CD300f* ^−/−^ mice were fed with high fat diet (HFD) for up to 12 weeks and their weight was monitored at the indicated time points (**A**,**C**). After ten (**B**) and twelve (**D**) weeks of HFD, the mice underwent glucose tolerance test (**B**,**D**). *Il5* ^*Tg*^ and *Il5* ^*Tg*^ */CD300f* ^−/−^ mice were fed with NCD or HFD for twelve weeks. Thereafter, the white adipose tissue (WAT) was obtained, photographed (**E**) and weighed (**F**). In addition, adipocyte size was assessed in H&E stained slides (**G**–**H**). Data in (**A–D**) are representative of n = 12; in (**E** and **G**) photographs are representative of 6/12 mice; In (**F** and **H**), n = 14; *p < 0.05, **p < 0.01, ***p < 0.001 as analyzed by two-way ANOVA followed by Tukey post-hoc test (**A**–**D**) and Student’s *t-test* (**F**–**H**).

To further explore the association between increased eosinophilia in *Cd300f* ^−/−^ mice and decreased weight gain and glucose metabolism, we subjected eight-week old *Il5* ^*Tg*^ and *Il5* ^*Tg*^ */Cd300f* ^−/−^ mice to a normal chow and high fat diet. Notably, glucose tolerance test following normal chow diet revealed no significant differences between *Il5* ^*Tg*^ and *Il5* ^*Tg*^ */Cd300f* ^−/−^ mice (Figure S8). Although *Il5* ^*Tg*^ mice were relatively protected from diet-induced obesity and glucose intolerance they still exhibited significantly increased weight gain and glucose intolerance in comparison with normal chow-fed mice or day 0 (Fig. 7A,B). In contrast, *Il5* ^*Tg*^ */Cd300f* ^−^/mice displayed markedly decreased weight accumulation in response to high fat diet (Fig. 7C). Protection from diet-induced weight gain was associated with substantially improved glucose tolerance testing (Fig. 7D). Moreover, WAT size (Fig. 7E) and weight (Fig. 7F) were substantially smaller in *Il5* ^*Tg*^ */Cd300f* ^−/−^ mice. Assessment of *Il5* ^*Tg*^ */Cd300f* ^−/−^ mice in metabolic cages showed that decreased weight gain in *Il5* ^*Tg*^ */Cd300f* ^−/−^ mice was not due to decreased food/water uptake, appetite or increased urination (S9). Importantly, although no differences were observed in weight gain between *Il5* ^*Tg*^ and *Il5* ^*Tg*^ */Cd300f* ^−/−^ mice in response to normal chow diet (S10), decreased adipose tissue weight and size was still observed (Fig. 7F). Consistent with decreased WAT size and weight, adipocyte size was smaller in the WAT of *Il5* ^*Tg*^ */Cd300f* ^−/−^ mice in comparison with *Il5* ^*Tg*^ mice (Fig. 7G,H).

## Discussion

The ability of eosinophils to migrate into the adipose tissue and modulate the activities of macrophages by secreting IL-4, has become a paradigm in adipose tissue homeostasis^37^. Nonetheless, pathways that regulate the recruitment and subsequent activation of eosinophils in the adipose tissue are largely unknown. In this study, we demonstrate that: a) CD300f is uniquely expressed by eosinophil progenitors; b) overexpression of IL-5 increases CD300f expression and baseline CD300f expression is partially dependent on IL-5; c) overexpression of IL-5 in the absence of CD300f results in a marked and specific increase in adipose tissue eosinophils, which were located in cellular foci and were accompanied by the accumulation of giant cells and macrophages; d) Specific accumulation of eosinophils in the adipose tissues of *Il5* ^*Tg*^ */Cd300f* ^−/−^ was associated with increased expression of a CD300f ligand in viable adipose tissue endothelial cells and elevated production of IL-4; e) Subsequently, increased IL-4 expression resulted in increased monocyte and macrophage proliferation consequently resulting in increased macrophage levels and induction of hallmark alternatively activated genes; f) CD300f regulated eosinophil and macrophage accumulation in the adipose tissue in an age-dependent fashion and was associated with decreased white adipose tissue size and weight as well as decreased weight gain and glucose intolerance after feeding with high fat diet. Finally, g) *Cd300f* ^−/−^ mice displayed a dramatic reduction in diet-induced weight gain and glucose intolerance in the presence of elevated IL-5 levels. Collectively, our data illustrate CD300f as a novel intrinsic counter-regulator of IL-5-driven adipose tissue eosinophil accumulation and IL-4 production, which governs diet-induced adipose tissue homeostasis and subsequent weight gain and glucose metabolism.

Notably, the finding that *Il5* ^*Tg*^ */Cd300f* ^−/−^ mice had increased alternatively activated macrophage accumulation in the adipose tissue, which was associated with decreased diet-induced weight gain and insulin resistance is likely an eosinophil-dependent phenomenon. This notion is based on several observations. We have recently shown that ILC2 cells (which have key roles in the adipose tissue)^4, 6^ and adipose tissue macrophages do not express CD300f^21, 32^. Furthermore, murine IL-5 receptor is uniquely expressed by eosinophils and B cells^38, 39^, and eosinophil-derived IL-4 has been recently shown to regulate macrophage polarization and subsequent glucose metabolism^3^. One of our striking observations was the specific increase in accumulation of eosinophils into the WAT and BAT of *Il5* ^*Tg*^ */Cd300f* ^−/−^ mice. This finding was of particular interest since the back of *Il5* ^*Tg*^ */Cd300f* ^−/−^ mice was extremely rigid (likely due to stiffness of the BAT) to a state that we could differentiate between *Il5* ^*Tg*^ */Cd300f* ^−/−^ and *Il5* ^*Tg*^ mice simply by identifying a dorsal patch of induration (100% prediction). Directly related, many obese individuals develop a humpback (commonly termed: Buffalo hump). Although the mechanisms for hump formation are unclear, excessive cortisol levels and excessive fat deposits have been implicated in this abnormal anatomical formation, at least in part. It is interesting that the mice developed fat accumulation which clinically resembled a “buffalo hump” but this cannot be simply explained as these same mice were protected from obesity and the presence of eosinophils in buffalo humps has not been examined. Nevertheless, our data suggest that IL-5, CD300f and eosinophils are integrated component of adipose tissue formation and may have implications for the type of dysregulated processes that involved in humpback formation.

*Il5* ^*Tg*^ mice display elevated eosinophilia in various organs such as the spleen, peritoneal cavity and GI tract^40^, which were not affected by the lack of *Cd300f*. This organ-specific phenomenon, is likely explained by two complementary findings. First, we demonstrate that the adipose tissue of *Il5* ^*Tg*^ */Cd300f* ^−/−^ mice displayed substantially increased expression of monocyte/macrophage-derived CCL24 (but not CCL11), which has been previously shown to regulate eosinophil migration to the gut and adipose tissue in an integrin-dependent fashion^3, 41, 42^. Moreover, our findings demonstrate that baseline IL-4 secretion was increased in *Cd300f* ^−/−^ eosinophils and that IL-4 secretion was further increased in *Cd300f* ^−/−^ eosinophils following activation with PMA. Whether CD300f negatively regulates IL-4 production from eosinophils in response to local factors in the adipose tissue is yet to be defined. Since IL-4 stimulates CCL24 production^43^, increased CCL24 production by macrophages is likely caused by the elevated levels of IL-4, which were present in the adipose tissue of *Il5* ^*Tg*^ */Cd300f* ^−/−^ mice. Second, we show that adipose tissue endothelial cells distinctly express a CD300f ligand, and that its expression is amplified in the presence of IL-5. We have recently shown that CD300f acts as a negative regulator of eosinophil chemotaxis towards CCL24 and CCL11 but not towards other chemo attractants such as LTB_4_^21^. Therefore, the finding that adipose tissue endothelial cells express ligands for CD300f places them at an interface to deliver inhibitory signals via CD300f to suppress eosinophil accumulation in response to adipose derived CCL24. Collectively, integration of these findings suggests a model where eosinophil accumulation in the adipose tissue is suppressed by CD300f upon binding to its ligand. In the absence of CD300f, the inhibitory signal towards IL-5-driven and CCL24-mediated accumulation of eosinophils is impaired. Thus, more eosinophils enter the adipose tissue where they secrete increased levels of IL-4. Subsequently, elevated IL-4 activates macrophages to secrete increased levels of CCL24, which further enhances eosinophil migration in a “vicious cycle”-like fashion.

Although *Cd300f* ^−/−^ mice displayed decreased weight gain and increased glucose uptake, these parameters were relatively modest especially in comparison with *Il5* ^*Tg*^ */Cd300f* ^−/−^ mice. The reason for this modest baseline effect is possibly due to additional functions that CD300f has in the regulation of immune responses by type 2 cytokines such as IL-4 and IL-33^32, 44^. We have recently shown that CD300f is physically associated with IL-4Rα and that it is necessary for IL-4-induced activation of multiple cells including macrophages and eosinophils. In fact, IL-4 stimulated *Cd300f* ^−/−^ macrophages display decreased activation of STAT6 and subsequently decreased induction of hallmark alternatively activated markers such as Relm-α and arginase1^44^. Thus, on one hand *Cd300f* ^−/−^ mice display elevated eosinophilia which secrete increased levels of IL-4 and on the other hand, *Cd300f* ^−/−^ macrophages may be less responsive to IL-4, which may cause them to promote obesity and glucose intolerance. This balance is substantially altered in the presence of IL-5, which enhances eosinophil accumulation and results in markedly elevated levels of IL-4 that can overcome the requirement for CD300f in IL-4-induced responses. In support of this notion, dose response experiments stimulating macrophages with IL-4 revealed that CD300f is required for IL-4-induced macrophage stimulation at low doses (1–20 ng/ml) but not at high doses (i.e. 50–100 ng/ml) (Munitz *et al*. data not shown). Therefore, increased levels of IL-4 in the adipose tissue of *Il5* ^*Tg*^ */Cd300f* ^−/−^ mice is likely capable of overcoming the requirement of CD300f in IL-4 induced responses. Hence, macrophages (which express low levels of CD300f) in *Il5* ^*Tg*^ */Cd300f* ^−/−^ mice can readily respond to IL-4 and are polarized into an alternatively activated state. This results in substantial protection from diet-induced weight gain and insulin resistance in *Il5* ^*Tg*^ */Cd300f* ^−/−^ mice in comparison with *CD300f* ^−/−^ mice. While we cannot exclude regulatory effects of CD300f on macrophages and/or monocytes it is important to note that under baseline conditions the expression of CD300f in the adipose tissue is exclusively confined to eosinophils^21^. Furthermore, macrophages and monocytes do not respond to IL-5. Thus, if such an effect is present, it is likely a secondary one.

Our study also highlights a key role for CD300f in IL-5-driven accumulation of adipose tissue eosinophils. This finding has therapeutic implications as the IL-5:IL-5Rα signaling pathway is now an entry point for a new class of emerging medicines^45^. Given the importance of IL-5-induced effects on eosinophils, it is prone to tight negative regulation in order to avoid hypereosinophilic responses. In this study, we show specific and restricted regulation of IL-5-driven response by CD300f in adipose tissue eosinophils. Nonetheless, CD300f can regulate the activities of various cells and has been implicated in multiple diseases including allergic diseases, colitis, brain injury and experimental autoimmune encephalomyelitis^46–49^. Specifically, the expression of CD300f was increased in various clinical conditions. For example, CD300f mRNA expression was increased in biopsies from pediatric Crohn’s disease patients. Furthermore, peripheral blood eosinophils from atopic individuals displayed elevated CD300f. In fact, CD300f regulated the development of experimental colitis and allergic diseases^21, 47, 49, 50^. Thus, our findings regarding the role of CD300f in the adipose tissue are likely translatable to human disease as well.

The regulatory function of CD300f on IL-5 appears to be relatively specific in regards to homing of eosinophils into the adipose tissue. CD300f likely acts by counter-regulation of specific IL-5 signaling events such as ERK and pAKT. It is important to note that our signaling studies bear the limitation that they were conducted in B cells that express IL-5 receptors and not eosinophils. Thus, it is possible that in eosinophils, CD300f may govern different pathways. Nonetheless, these studies support our overall observation that CD300f is capable of regulating IL-5-induced effects in the signaling level as well. The finding that CD300f can regulate distinct IL-5-driven responses and signaling cascades is consistent with findings regarding additional receptors expressed by eosinophils, which can regulate only a fraction of IL-5-induced responses in eosinophils. For example, eosinophils that were isolated from mice that lack the signaling domain of SIRPα (*Sirp1a Cyto* ^−/−^ mice) showed no developmental defects in eosinophils and only displayed reduced viability and degranulation, with specific effects in the small intestine^51^. Furthermore, the inhibitory receptor PIR-B served as a permissive checkpoint for IL-5-induced development of eosinophils in the bone marrow by suppressing the pro-apoptotic activities of PIR-A, which were mediated by the Grb2-Erk-Bim pathway^22^. These effects were exclusive to the bone marrow but not peripheral organs. Finally, CD300a, the additional ITIM-bearing receptor belonging to the CD300-Ig superfamily negatively regulates human eosinophil survival in response to GM-CSF and IL-5^52^. Thus, it appears that IL-5-induced eosinophil responses are differentially regulated by ITIM-bearing receptors in a tissue-dependent fashion that is likely based on the availability of ligands and or receptor expression. As anti-IL-5 therapy is now in the clinic, it will be interesting to assess the expression and function of ITIM-bearing receptors in eosinophils from human patients before and after treatment and to assess whether any metabolic (e.g. obesity, diabetes) alterations occur in individuals chronically treated with anti-IL-5 therapy.

Despite its inhibitory activity towards IL-5 induced responses *in vivo*, the *in vitro* experiments show that CD300f differentially regulates IL-5-induced signaling cascades. On one hand, CD300f was capable of suppressing IL-5-dependent ERK activation and on the other hand it induced phosphorylation of AKT. The finding that CD300f can elicit both inhibitory and activating signals is consistent with the presence of intracellular domains that contain two ITIM motifs (which mediate inhibition), one immunoreceptor tyrosine-base switch motif (ITSM)^53^ and a p85α phosphoinositide 3-kinase-binding motif (which mediates co-activation). Indeed, various studies have demonstrated dual and opposing functions for CD300f. For instance, CD300f acts as a functional binding partner and co-receptor for IL-4Rα, can co-localize with ST2 and can promote phagocytosis^20, 32, 44^. In contrast, CD300f can suppress cellular chemotaxis and toll-like receptor signaling^21, 54, 55^. Furthermore, CD300f has been shown to have opposing activation and inhibitory functions in mast cell activation^48, 49^. Interestingly, our data demonstrate a novel finding with respect to CD300f signaling. To date, the co-activating or inhibitory activities of CD300f were shown in response to different stimuli, which either trigger co-activation of inhibition. We show that in response to the same stimulus (i.e. IL-5), CD300f is capable of inhibitory (e.g. ERK suppression) and co-activating (e.g. AKT activation) signals simultaneously. Consequently, the overall inhibitory effect of CD300f in respect to IL-5, is likely due to an intracellular balance between differential signaling cascades.

In summary, we provide evidence that CD300f is an intrinsic regulator of IL-5-induced eosinophil responses with specific effects in the adipose tissue. These findings provide new insights regarding the molecular pathways fine tuning the activities of adipose tissue eosinophilia and have significant implications in promoting metabolic homeostasis through the eosinophil-macrophage axis.

## Methods

### Mice

WT C57BL/6 mice were obtained from Harlan Laboratories (Rehovot, IL) and maintained in-house. Generation of *Cd300f* ^−/−^ and *Il5* ^−/−^ mice has been previously described^46, 56^. *Cd300a* ^−/−^ mice (B6N.129S5-Cd300atm1Lex/Mmcd, ID 032182-UCD) were obtained from the Mutant Mouse Regional Resource Center (MMRC, University of California, Davis, CA; originally donated by Genentech) and backcrossed to C57BL/6 mice for 10 generations^21^. CD3-IL-5 transgenic mice (NJ.1638, *Il5* ^*Tg*^) mice were kindly provided by Dr. Jamie Lee (Mayo Clinic, Scottsdale, AZ)^40^. *Il5* ^*Tg*^ were crossed with *Cd300f* ^−/−^ mice for two generations to form the *Il5* ^*Tg*^/*Cd300f* ^−/−^ line (both on the C57Bl/6 background). All experiments were reviewed and approved by the Animal Care Committee of Tel Aviv University (Number M-13-029, M-13-30), and were performed in accordance with its regulations and guidelines regarding the care and use of animals for experimental procedures. All of the experiments were conducted in the specific pathogen free facilities of the Tel Aviv University. In all experiments, age-, weight-, and sex-matched mice were used.

### BM-derived eosinophil culture

Eosinophils were grown from the BM of WT and *Cd300f* ^−/−^ mice with modifications based on a prior report^57^. Briefly, BM cells were harvested and loaded on a histopaque gradient (Sigma, Rehovot, IL). The low-density BM cells were collected and enriched for lineage negative cells using negative selection with magnetic beads linked to a specific antibody cocktail (Miltenyi Biotech, Germany) according to manufacturer’s instructions. The lineage negative cells were then cultured in the presence of 100 ug/ml SCF and Flt3L for 4 days. Thereafter, the medium was replaced with 10 μg/ml IL-5 for the rest of the culture (up to day 13). All cytokines were purchased from Peprotech (Rehovot, IL).

### Quantitative (q) PCR

Total RNA was isolated using Trizol (Invitrogen, Grand Island, NY) and subjected to qPCR analysis. RNA samples were subjected to reverse transcription analysis using iScript cDNA synthesis kit (obtained from Bio-Rad, Hercules, CA) according to manufacturer’s instructions. qPCR analysis was performed using the CFX96 system (Bio-Rad laboratories, Hercules, CA) in conjunction with the ready-to-use iQ SYBR Green Supermix (obtained from Bio-Rad, Hercules, CA). Results were normalized to *Hprt* cDNA. The primers that were used in this study were as follows:

*Hprt*: GTAATGATCAGTCAACGGGGGAC, CCAGCAAGCTTGCAACCTTAACCA

*Il4*: TCAACCCCCAGCTAGTTG, TGTTCTTCGTTGCTGTGAG

*Retnla*: CCCTCCACTGTAACGAAGACTC, CACACCCAGTAGCAGTCATCC

CCL11 (*Ccl11*): CACGGTCACTTCCTTCACG, TGGGGATCTTCTTACTGGTA

CCL24 (*Ccl24*): TGTGACCATCCCCTCATCTGC, AAACCTCGGTGCTATTGCGCG

Chil3 (*Chil3l*): GAACACTGAGCTAAAAACTCTCCTG, GAGACCATGGCACTGAACG

*Arg1*: GAATCTGCATGGGCAACC, GAATCCTGGTACATCTGGGAAC

*Ccl2*: CCTGTCATGCTTCTGGGCCTGC, GGGGCGTTAACTGCATCTGGCTG

### Affymetrix cDNA microarray

RNA was isolated using Trizol (Invitrogen, Grand Island, NY) according to manufacturer’s instructions. RNA was then amplified, fragmented and labeled using the Ovation Pico WTA System V2 and the Encore Biotin Module (obtained from Nugen, San Carlos, CA). Mouse Affymetrix (Santa Clara, CA) microarrays (2.1 ST GeneChip) were performed and analyzed using established protocols of the Tel-Aviv University Bioinformatics Unit and according to the manufacturer’s instructions^58^.

### Enzyme-linked immunosorbent assay (ELISA)

Cytokines were measured by enzyme-linked immunosorbent assay according to the manufacturer’s instructions. CCL11 (Eotaxin-1) and CCL24 (Eotaxin-2) ELISA kits were purchased from R&D Systems (Minneapolis, MN). IL-4 ELISA kit was purchased from BioLegend (San Diego, CA). CCL2 was obtained from Peprotech (Rechovot, IL) and Relm-α was detected as previously described^59^. Lower detection limits for these assays were 15.625 pg/ml (CCL11) and 31.25 pg/ml (CCL24, CCL2 and Relm-α).

### Preparation of tissue single cell suspension

Abdominal and neck adipose tissue were excised and incubated in 1.5 mg/ml collagenase, 0.75 mg/ml hyaluronidase and 0.1 mg/ml DNase I (Sigma, Rehovot, IL) containing DMEM (Gibco, Invitrogen, Grand Island, NY) with 2% fetal bovine serum (FBS) for 1 hour at 37 °C. The suspension was vortexed and strained. Colon and jejunum was washed with phosphate-buffered saline (PBS), cut into small pieces and incubated in Hank’s Balanced Salt Solution (HBSS, Biological industries, IL) supplemented with 5% FBS, 2 mM EDTA and 1 mM DTT, for 40 minutes at 37 °C. Afterwards, the colon pieces were incubated in PBS with calcium chloride and magnesium chloride, supplemented with 5% FBS, 1 mg/ml collagenase A (Roche, Germany) and 0.1 mg/ml DNase I (Sigma, Rehovot, IL), for 40 minutes at 37 °C. The suspension was vortexed, strained and centrifuged to receive single cell suspension of lamina propria cells. Peritoneal lavage was extracted by penetrating and flushing the peritoneal cavity with a 21G needle joined to a 10 ml PBS containing syringe and drawing back the cell containing fluid. Spleen was crushed against a 70 μm cell strainer sunk in PBS. BM cells were extracted as described above. Blood sample were collected from the eye using cannula and heparin-coated tube while the mouse is sedated with a non-lethal dose to isoflurane 99.9%.

### Tissue eosinophil collection

Single cell suspension was obtained from abdominal adipose tissue or bone marrow as described above. The cells were then enriched for eosinophils using positive selection with magnetic beads (Miltenyi Biotech, Germany) linked to anti-Siglec-F antibody according to manufacturer’s instructions.

### Flow cytometry of extracellular markers

Single cell suspensions were obtained from the desired organ or culture and centrifuged. The cells were stained using the following antibodies: anti-CD125 (IL-5Ra)-Alexa Fluor 488, anti-Siglec-F-PE, anti- Siglec-F-Brilliant violet 420, anti-CD135- PerCP eFluor 710 (BD Bioscience, San Jose, CA), anti-CD11b-PerCP-Cy5.5, anti-CD45-PE-CY7, anti-CD45-APC eFluor 780, anti-c-kit (CD117)-APC eFluor 780, Sca-1 (Ly6A/E)-PE, anti-CD34-FITC, anti-CD34-Alexa Fluor 700, anti-F4/80-PE, anti-F4/80- APC-CY7, Rat IgG2a, Rat IgG1-Alexa Fluor 488 (eBioscience, San Diego, CA), anti-CCR3-FITC, anti-CD300a (R&D Systems, Minneapolis, MN), anti-Ly6G-FITC, anti-LY6C-PE-CY7, anti-Ly6G-Alexa Fluor 647, anti-CD16/32- PerCP-Cy5.5, Lineage cocktail-Pacific Blue (Cocktail includes anti-mouse CD3, clone 17A2; anti-mouse Ly-6G/Ly-6C, clone RB6-8C5; anti-mouse CD11b, clone M1/70; anti-mouse CD45R/B220, clone RA3-6B2; anti-mouse TER-119/Erythroid cells, clone Ter-119), Armenian Hamster IgG (BioLegend, San Diego, CA), anti-CD300f (Kindley provided by Dr. Menno Van-Lookeren, Genetech), Goat anti-Armenian Hamster IgG-Alexa Fluor 647, Goat anti-Armenian Hamster IgG-Alexa Fluor 488, Goat anti-Rat IgG-DyLight 649 (Jackson ImmunoResearch, West Grove, PA), anti-CD31-PE-CY7 (Peprotech, Rehovot, IL) and DAPI (Sigma, Rehovot, IL). The cells were acquired by a Beckman-Coulter Gallios flow cytometer and analyzed by Kaluza (Beckman-Coulter, Brea, CA). Cell counts were performed by adding Flow-Count^TM^ Fluorospheres (Beckman-Coulter, Brea, CA) according to manufacturer’s instructions.

### Cell proliferation

EdU incorporation was performed by injecting 0.5 mg EdU IP and sacrificing the animals 24 hours later. Cell proliferation was assessed according to Click-iT® Edu Flow cytometry assay kit Alexa Fluor 647 (Invitrogen, Grand Island, NY) manufacturer’s instructions. The cells were acquired by a Beckman-Coulter Gallios flow cytometer and analyzed by Kaluza (Beckman-Coulter, Brea, CA).

### Annexin-V apoptosis assay

Apoptotic cells were detected using Annexin V Apoptosis Detection Set PE-CY7 (eBioscience, San Diego, CA) according to manufacturer’s instructions. The cells were acquired by a Beckman-Coulter Gallios flow cytometer and analyzed by Kaluza (Beckman-Coulter, Brea, CA).

### CD300f ligand staining

CD300f-IgG1-Fc-fusion protein was generated as previously described (Xi, 2010 #1). Single cell suspension from abdominal adipose or lung tissues was incubated with 50 μg/ml CD300f-Fc-fusion or appropriate amount of mouse IgG1 control, followed by incubation with Goat anti-mouse Alexa Fluor 488 (Jackson, ImmunoResearch, West Grove, PA). The cells were acquired by a Beckman-Coulter Gallios flow cytometer and analyzed by Kaluza (Beckman-Coulter, Brea, CA).

### Protein secretion from adipose tissue samples

Abdominal and neck adipose tissue were excised and adjusted to 50 mg and incubated in 48 well plate (BD Falcon) in IMDM (Gibco, Invitrogen, Grand Island, NY) medium with 10% FBS, 1% penicillin-streptomycin (Biological industries, IL) and 2 mM glutamine (Gibco, Invitrogen, Grand Island, NY) for 24 hours. Supernatants were collected and kept at −80 °C until assessed for cytokines by ELISA.

### Viral infection of I.29 cells

I.29 (B lymphoma) cells were cultured in PRMI-1640 medium supplemented with 10% FBS, 1% penicillin-streptomycin (Biological industries), 2 mM glutamine, 55 μM mercaptoethanol (Invitrogen, Grand Island, NY) and 5% CO_2_. pCDH-puro expression vectors encoding WT CD300f or empty vector were generated and lentivirus particles obtained as previously described (ref). Cells (2 × 10^5^) were infected using polybrene in media containing 50% viral particles. 1 μg/ml puromycin was supplemented to cell culture medium for at least 2 weeks to yield a stable infected line with over 95% infected cells. All experiments were later performed in the absence puromycin.

### Phospho-flow assay

CD300f or empty vector expressing I.29 cells were activated with 100 ng/ml IL-5 (Peprotech, Rehovot, IL) kinetically (0–45 minutes). Subsequently, the cells were fixated with 3.7% formaldehyde and stained with Alexa Fluor 647 conjugated ERK1/2 antibody (Cell Signaling, Danvers, MA) in saponin (Invitrogen, Grand Island, NY) supplemented buffer for intracellular staining. IL-5Rα expression was determined using Alexa Fluor 488 conjugated IL-5Ra antibody (BD Bioscience, San Jose, CA).

### Western blot

CD300f expressing I.29 cells were activated with 100 ng/ml IL-5 (Peprotech, Rehovot, IL) for 0–10 minutes. Ice-cold lysis buffer containing 25 mM Tris-HCl pH 7.4, 150 mM NaCl, 1% NP-40, 1 mM EDTA, 5% glycerol (Pierce, Thermo Fisher Scientific, Waltham, MA) was then added to the cells. Cell lysates were directly mixed in SDS-PAGE loading buffer, resolved by SDS-PAGE, and transferred onto PVDF membrane (Millipore). Membranes were blocked with 5% skim milk in TBST (20 mM Tris pH 7.4, 150 mM NaCl, 0.05% Tween-20) for 1 h and then probed with anti-phospho-AKT, anti-AKT (Cell Signaling, Danvers, MA) overnight, followed by HRP-conjugated anti-rabbit secondary antibody or HRP-conjugated anti-Armenian hamster antibody (Jackson ImmunoResearch, West Grove, PA) for 1 h. Antibodies were diluted in either 2.5% skim milk in or 5% BSA in TBST according to manufacturer’s instructions. Blots were developed with Luminata Crescendo Western HRP Substrate and visualized on using the fusion pulse 6 chemiluminescent instrument (Analis, Belgium).

### Immunohistochemistry

Anti-MBP antibody was kindly provided by Dr. Jamie Lee, (Mayo clinic, Scottsdale, AZ) and immunohistochemistry performed as previously described using the ABC and DAB kit^21^. Counterstain was performed with nuclear fast red (obtained from Sigma, St. Louis, MO). H&E staining was performed by Patho-Lab diagnostics (Rehovot, Israel). Images were captured using an Olympus AX70 fluorescent microscope (Center Valley, PA, USA) equipped with a DP72 camera.

### High fat diet model

Male mice (aged 6 weeks) were subjected to either 10% fat normal chaw diet or 60% fat diet (Envigo, UK) for at least 12 weeks. Water and food were changed weekly along with weight monitoring.

### Glucose tolerance test

Mice were fed for 16 hours prior to measurement. Dextrose (Sigma, Rehovot, IL) was IP administrated at 2-gram dextrose per kg body weight. Blood glucose levels were measured at time 0 (before administration) and 15, 30, 60, 90 and 120 minutes post injection using a glucometer and counter meter strips (Perrigo, Bnei-Brak, IL).

### Metabolic cages

Each male mouse (aged 6 weeks) was placed in metabolic cages (Braintree Scientific, Inc. Braintree, MA) with premeasured food weight and water volume. After 24 hours, feces, urine and remaining food and water were collected and measured.

### Statistical analysis

Data were analyzed by analysis of variance followed by Tukey post hoc test or Student’s t-test using GraphPad Prism 5 (obtained from GraphPad, San Diego, CA). Data are presented as mean ± SEM, and values of P < 0.05 were considered statistically significant.

## Electronic supplementary material

Supplementary data
