# Supplementary material for: CD300f:IL-5 cross-talk inhibits adipose tissue eosinophil homing and subsequent IL-4 production
Source: Sci Rep. 2017 Jul 19;7:5922. doi: 10.1038/s41598-017-06397-4 (PMC5517555; doi:10.1038/s41598-017-06397-4)
Supplement: Supplementary file 1 — Supplementary data [file 41598_2017_6397_MOESM1_ESM.docx]

**Supplementary Data online**

**CD300f:IL-5 cross-talk inhibits adipose tissue eosinophil homing and subsequent IL-4 production**

Perri Rozenberg^1^, Reichman Hadar^1^, Israel Zab-Bar^1^, Itan Michal^1^, Pasmanik-Chor Metsada^2^, Carine Bouffi^3^, Udi Qimron^1^, Ido Bachelet^4^, Patricia C. Fulkerson^3^, Marc E. Rothenberg^3^, Ariel Munitz^1,*^

^1^ Department of Clinical Microbiology and Imm­­­unology, The Sackler School of Medicine, Tel-Aviv University, Ramat Aviv 69978, Israel.

^2^ Bioinformatics Unit, George S. Wise Faculty of Life Sciences, Tel Aviv University, Tel Aviv, 64239, Israel

^3^ Division of Allergy and Immunology, Cincinnati Children’s Hospital Medical Center, 3333 Burnet Avenue, Cincinnati, OH, 45229, USA

^4^ Augmanity Nano LTD. Rehovot, Israel

**Running Title:** Adipose Tissue Eosinphils

*Corresponding author: Ariel Munitz, PhD, Department of Clinical Microbiology and Immunology, The Sackler School of Medicine, Tel-Aviv University, Ramat Aviv 69978, Israel. Tel. (Office): +972-3-640-7636, Fax: +972-3-640-9160, e-mail: arielm@post.tau.ac.il.

**Figure legends**

**Figure S1. Gating strategy for identification of bone marrow progenitor cells**

Total bone marrow cells were obtained from wild type mice and stained with the indicated antibodies for the identification of hematopoietic stem vcells (HSCs), multipotent progenitors (MPPs), megakaryocyte-erythroid progenitors (MEPs), common myeloid progenitors (CMPs), granulocyte-macrophage progenitors (GMPs) and eosinophil progenitors (EoPs) (A-B). Data are representative density plots of n= 5.

**Figure S2. Expression of CD300a in hematopoietic progenitor cells and mature eosinophils**

The expression of CD300a was assessed in bone marrow hematopoietic stem cells (HSCs, CD45^+^/Lin^-^/Sca-1^-^/c-kit^+^/CD135^-^), multipotent progenitors (MPPs, CD45^+^/Lin^-^/Sca-1^-^/c-kit^+^/CD135^-^), myeloid-erythrocyte progenitors (MEPs, CD45^+^/Lin^-^/Sca-1^+^/c-kit^+^/CD34^-^/CD32/16^+^), common myeloid progenitors (CMPs, CD45^+^/Lin^-^/Sca-1^+^/c-kit^+^/CD34^+^/CD32/16^-^), granulocyte-macrophage progenitor (GMPs, CD45^+^/Lin^-^/Sca-1^+^/c-kit^+^/CD34^+^/CD16^+^) and eosinophil progenitors (EoPs, CD45^+^/Lin^-^/Sca-1^+^/c-kit^+^/CD34^int^/IL-5Rα^+^) (A). Moreover, the expression of CD300a was assessed in eosinophils from the indicated organs that were obtained from WT and hypereosinophilic *Il5* transgenic (*Il5^Tg^*) mice (B). Data for (A) are representative of n=5 mice, for (B), n=6 mice.

**Figure S3. Microarray analysis of primary bone marrow eosinophils from *Il5^Tg^* and *Il5^Tg^/Cd300f^-/-^* mice**

RNA was obtained from primary bone marrow eosinophils that were sorted from *Il5^Tg^* and *Il5^Tg^/Cd300f^-/-^* mice and subjected to microarray analysis. Heat plot and hierarchical clustering of the microarray data is shown. Each lane represents one mouse.

**Figure S4. CD300f does not regulate IL-5-induced eosinophil differentiation**

Bone marrow-derived eosinophils were grown in vitro in the presence of IL-5 for up to 14 days. During the culture, total cell number (A), cellular proliferation (B) and apoptosis (C) were monitored. In addition, the level of total Siglec-F^+^ cells at the end of the culture was determined (D). Data are from n=3 experiments.

**Figure S5. Expression of CCL11 and CCL2 in the white and brown adipose tissue of *Il5^Tg^/Cd300f^-/-^* mice**

The mRNA expression of *Ccl11* and *Ccl2* was determined in the white and brown adipose tissue (WAT and BAT, respectively) of wild type (WT), *Cd300f^-/-^*, *Il5^Tg^* and *Il5^Tg^/Cd300f^-/-^* mice by quantitative PCR and normalized to the house keeping gene hypoxanthine-guanine phosphoribosyltransferase (*Hprt*) (A-B and D).

**Figure S6. Baseline metabolic analysis of wild type and *Cd300f^-/-^* mice**

Wild type (WT) and *Cd300f^-/-^* mice were held in metabolic cages for twenty four hours and monitored for baseline metabolic features including food uptake (A), percent of food uptake from body weight (B), feces (C) urine (D) and water uptake (E). Data are from n=10 mice per group; ns- non significant, **- p< 0.01 001 as analyzed by Student’s *t-test*.

**Figure S7. Weight gain and glucose tolerance of wild type and *Cd300f^-/-^* mice in response to normal chow diet**

Wild type (WT), *CD300f^-/-^* mice were fed with normal chow diet (NCD) for up to seven weeks and their weight was monitored at the indicated time points (A). After seven weeks of NCD, the mice underwent glucose tolerance test (B). Data are from n=10 mice per group.

**Figure S8. Glucose tolerance of *Il5^Tg^* and *Il5^Tg^/Cd300f^-/-^* mice in response to normal chow diet**

*Il5^Tg^* and *Il5^Tg^/Cd300f^-/-^* mice were fed with normal chow diet (NCD). After seven weeks of NCD, the mice underwent glucose tolerance test (B). Data are from n=10 mice per group; *- p< 0.05 as analyzed by two-way ANOVA followed by Tukey post-hoc test (E-F)..

**Figure S9. Baseline metabolic analysis of *Il5^Tg^* and *Il5^Tg^/Cd300f^-/-^* mice**

*Il5^Tg^* and *Il5^Tg^/Cd300f^-/-^* mice were held in metabolic cages for twenty-four hours and monitored for baseline metabolic features including food uptake (A), percent of food uptake from body weight (B), feces (C) urine (D) and water uptake (E). Data are from n=10 mice per group; ns- non significant.

**Figure S10. Weight gain of *Il5^Tg^* and *Il5^Tg^/Cd300f^-/-^* mice in response to normal chow diet**

*Il5^Tg^* and *Il5^Tg^/Cd300f^-/-^* mice were fed with normal chow diet (NCD) for up to seven weeks and their weight was monitored at the indicated time points. Data are from n=10 mice per group.

**Figures**

**Figure S1**

**
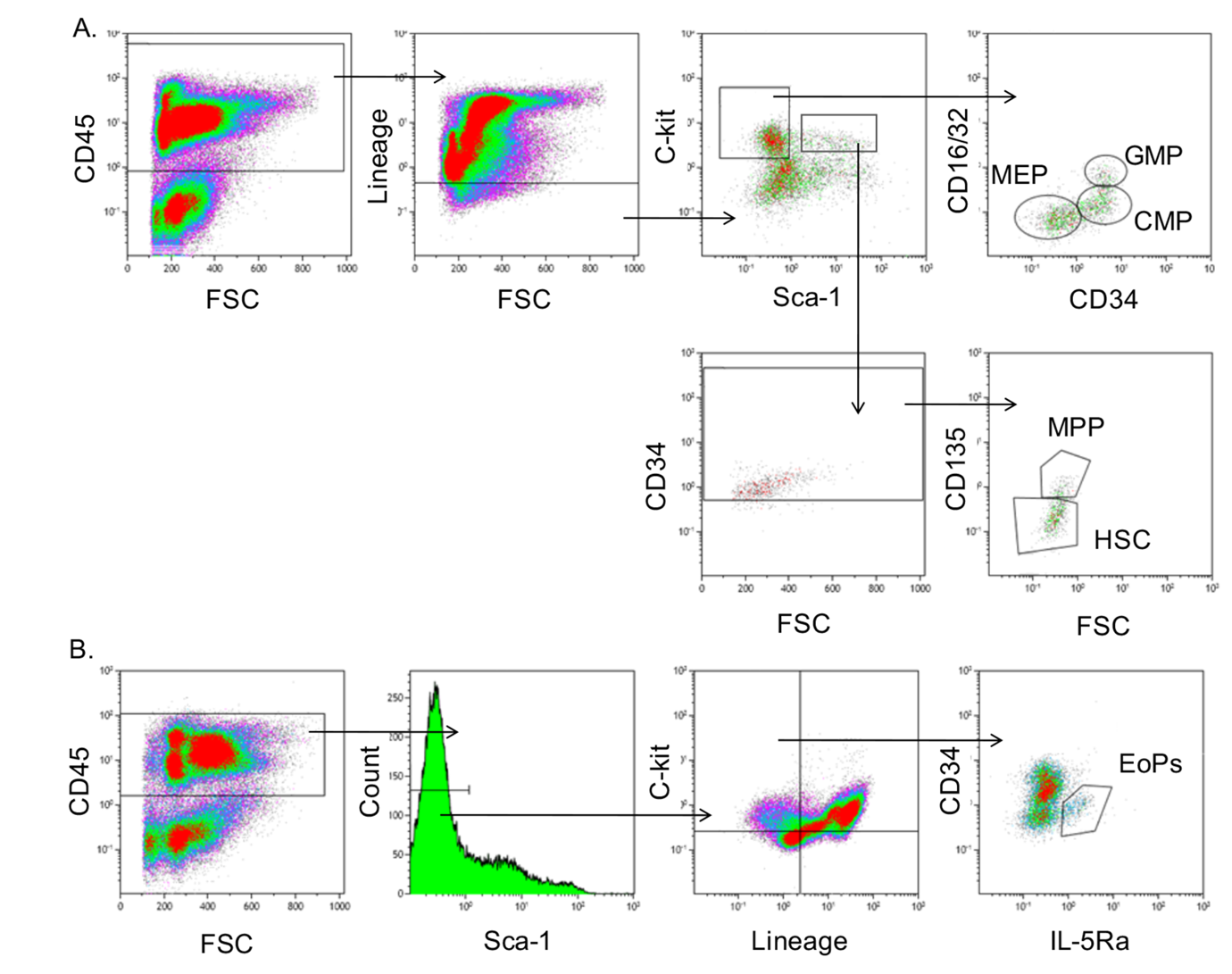
**

**Figure S2**

**
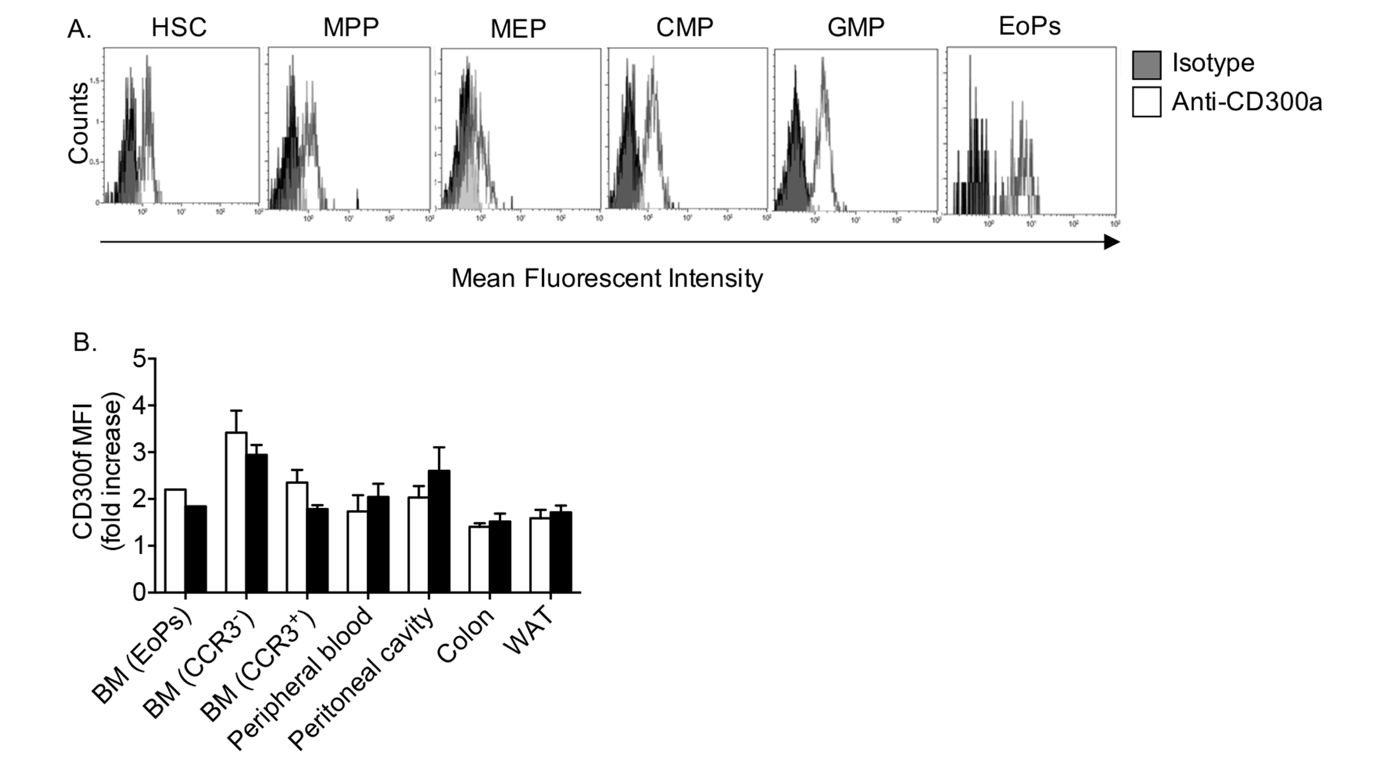
**

**Figure S3**

**
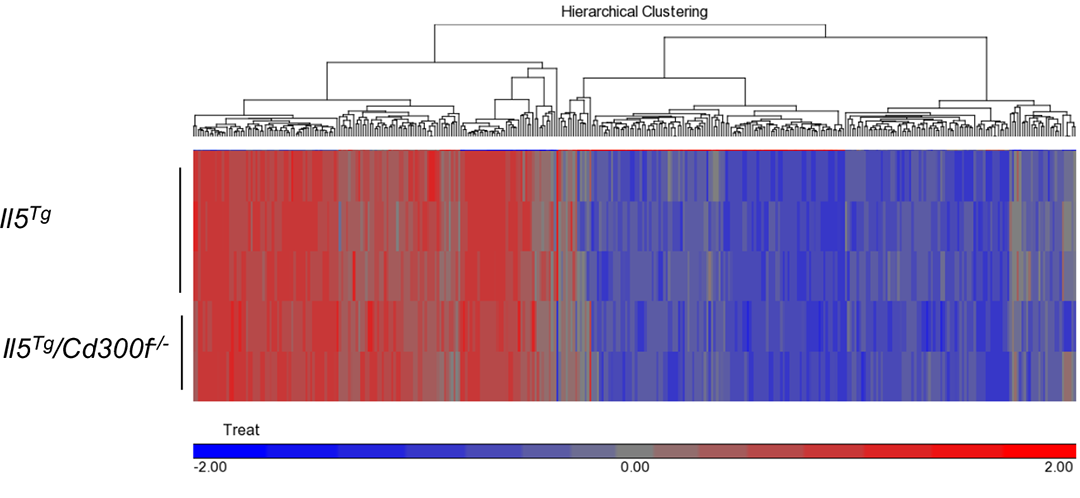
**

**Figure S4**

**
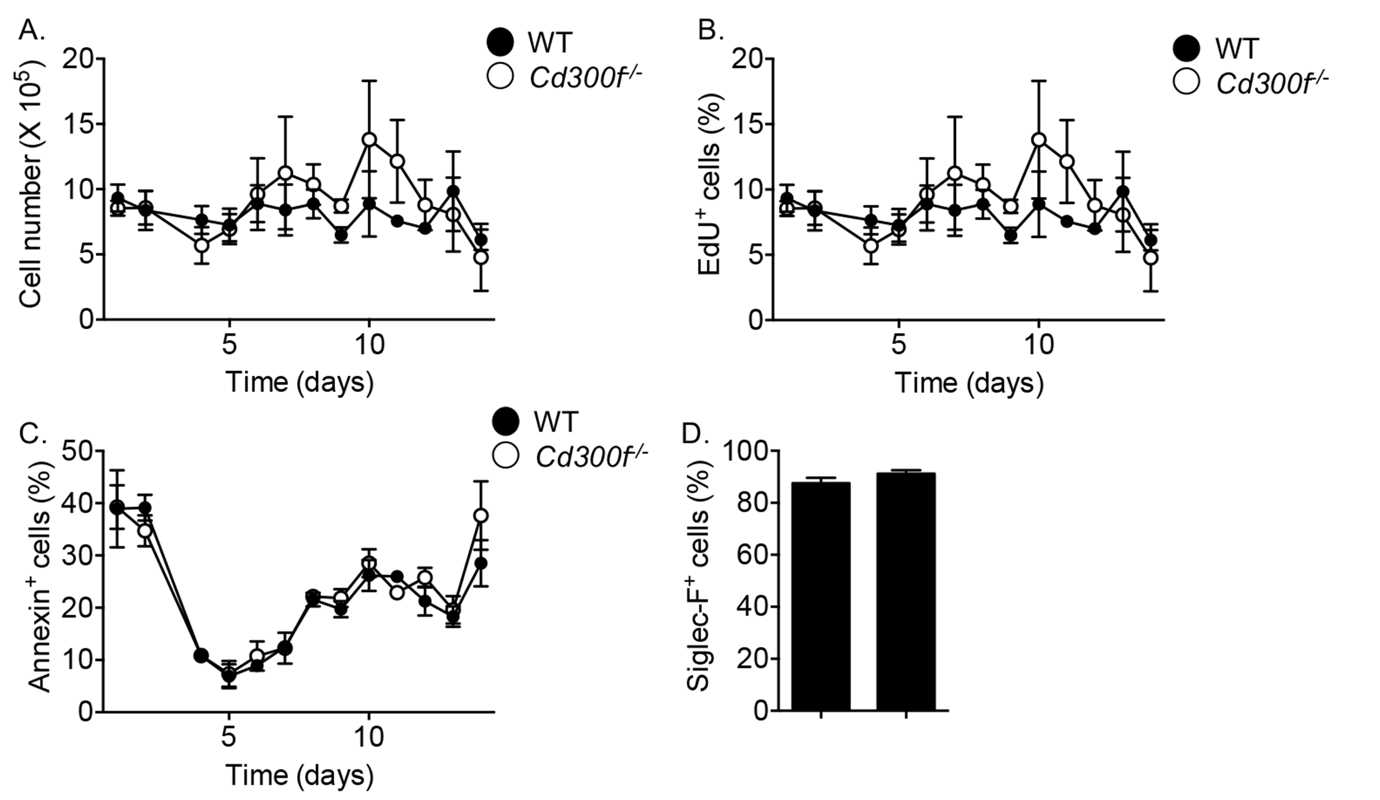
**

**Figure S5**

**
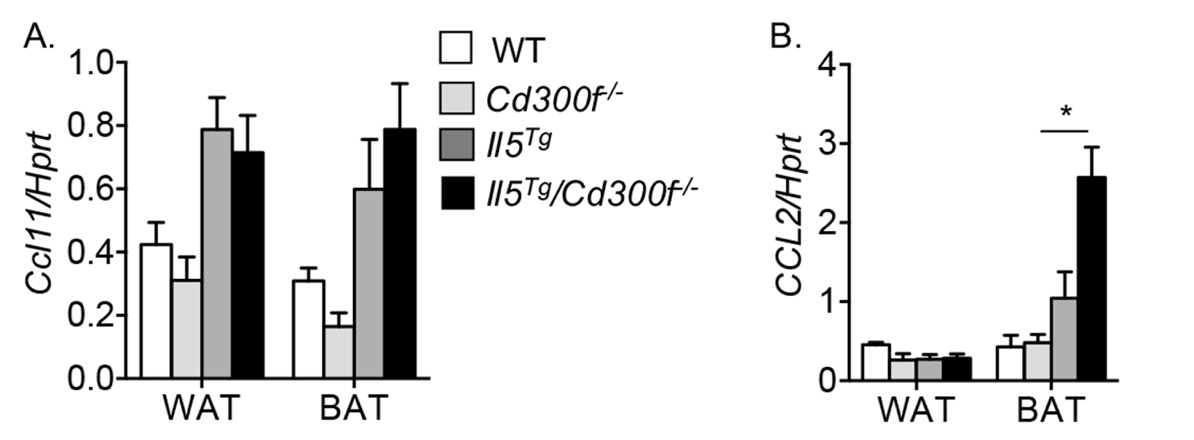
**

**Figure S6**

**
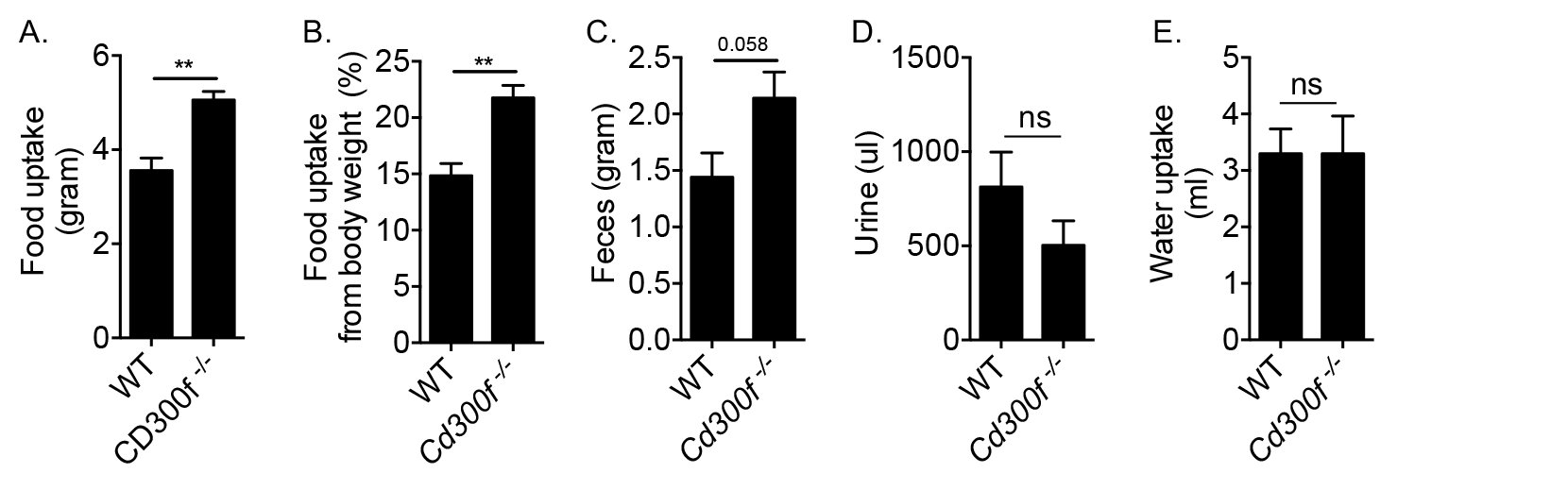
**

**Figure S7**

**
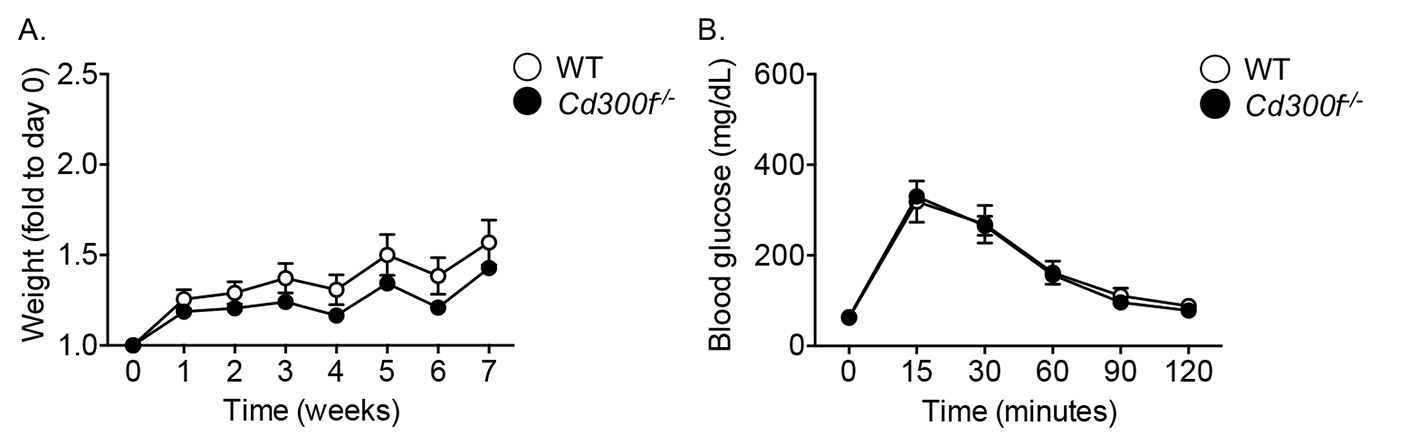
**

**Figure S8**

**
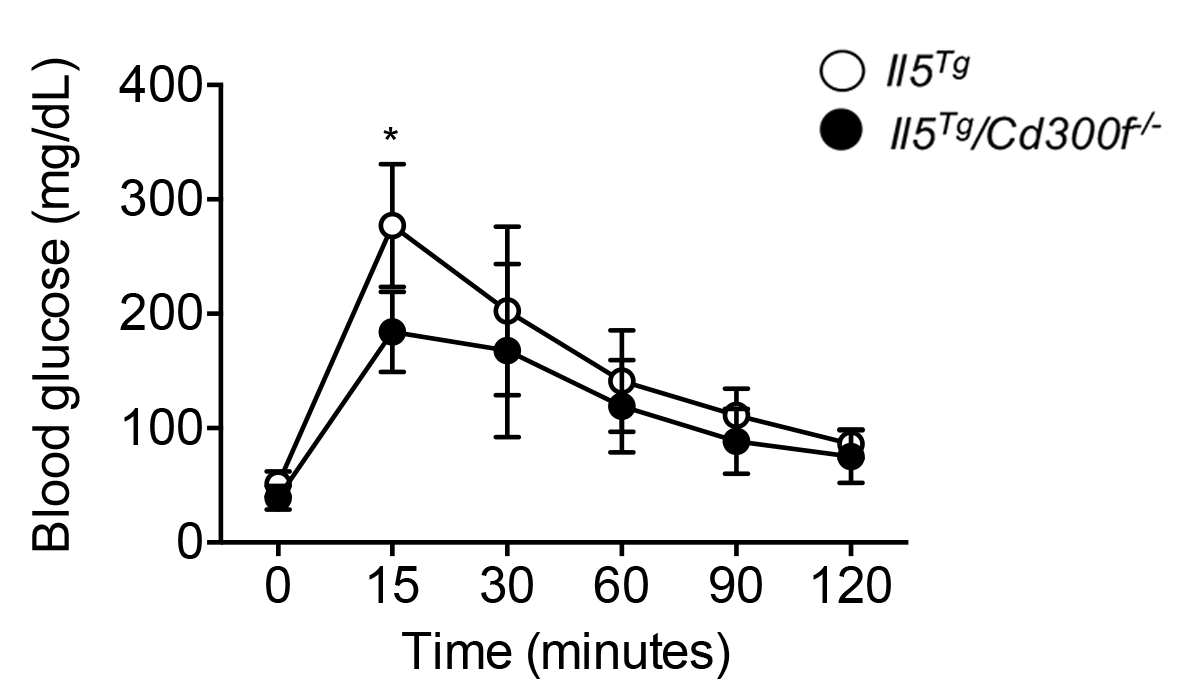
**

**Figure S9**

**
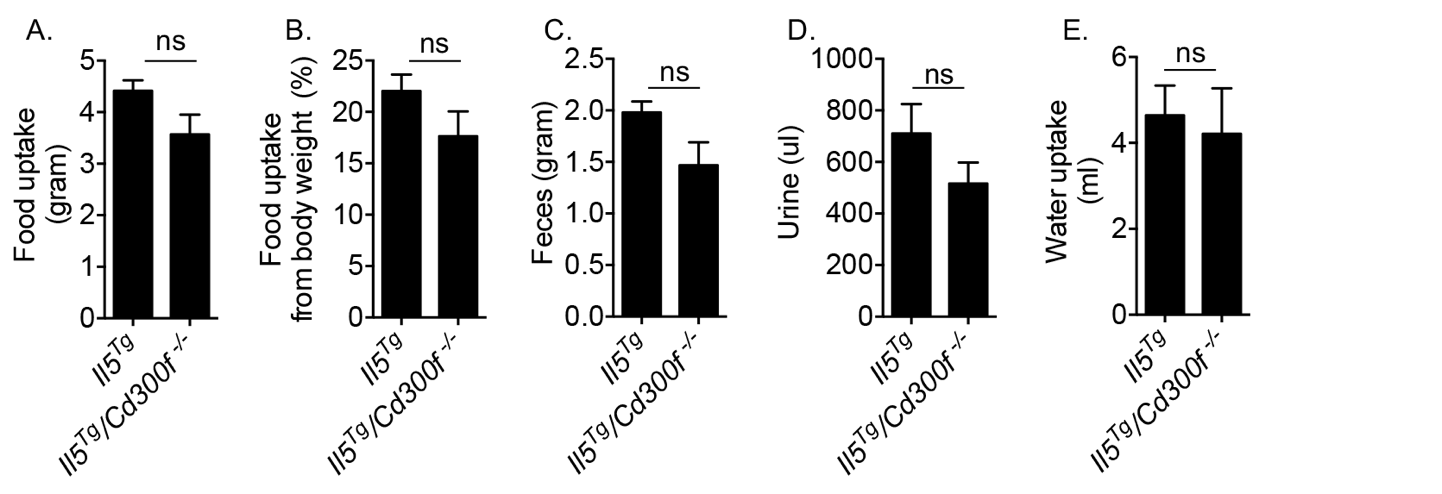
**

**Figure S10**

**
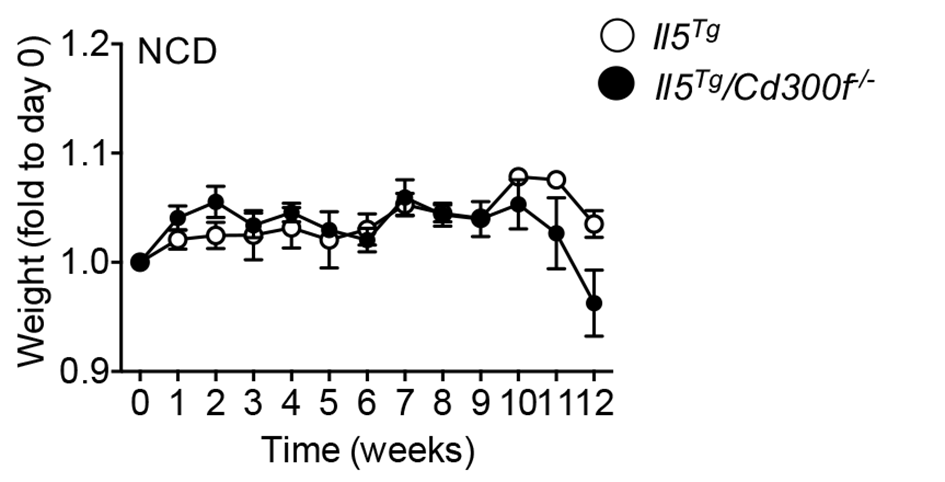
**
